# Acute high-fat diet impairs macrophage-supported intestinal damage resolution

**DOI:** 10.1172/jci.insight.164489

**Published:** 2023-02-08

**Authors:** Andrea A. Hill, Myunghoo Kim, Daniel F. Zegarra-Ruiz, Lin-Chun Chang, Kendra Norwood, Adrien Assié, Wan-Jung H. Wu, Michael C. Renfroe, Hyo Wong Song, Angela M. Major, Buck S. Samuel, Joseph M. Hyser, Randy S. Longman, Gretchen E. Diehl

**Affiliations:** 1 Department of Pathology and Immunology and; 2 Alkek Center for Metagenomics and Microbiome Research and the Department of Molecular Virology and Microbiology, Baylor College of Medicine, Houston, Texas, USA.; 3 Immunology Program of the Sloan Kettering Institute, Memorial Sloan Kettering Cancer Center, New York, New York, USA.; 4 Jill Roberts Institute for Research in IBD and Jill Roberts Center for IBD, Weill Cornell Medicine, New York, New York, USA.

**Keywords:** Immunology, Macrophages, Neutrophils

## Abstract

Chronic exposure to high-fat diets (HFD) worsens intestinal disease pathology, but acute effects of HFD in tissue damage remain unclear. Here, we used short-term HFD feeding in a model of intestinal injury and found sustained damage with increased cecal dead neutrophil accumulation, along with dietary lipid accumulation. Neutrophil depletion rescued enhanced pathology. Macrophages from HFD-treated mice showed reduced capacity to engulf dead neutrophils. Macrophage clearance of dead neutrophils activates critical barrier repair and antiinflammatory pathways, including IL-10, which was lost after acute HFD feeding and intestinal injury. IL-10 overexpression restored intestinal repair after HFD feeding and intestinal injury. Macrophage exposure to lipids from the HFD prevented tethering and uptake of apoptotic cells and *Il10* induction. Milk fat globule-EGF factor 8 (MFGE8) is a bridging molecule that facilitates macrophage uptake of dead cells. MFGE8 also facilitates lipid uptake, and we demonstrate that dietary lipids interfere with MFGE8-mediated macrophage apoptotic neutrophil uptake and subsequent *Il10* production. Our findings demonstrate that HFD promotes intestinal pathology by interfering with macrophage clearance of dead neutrophils, leading to unresolved tissue damage.

## Graphical abstract

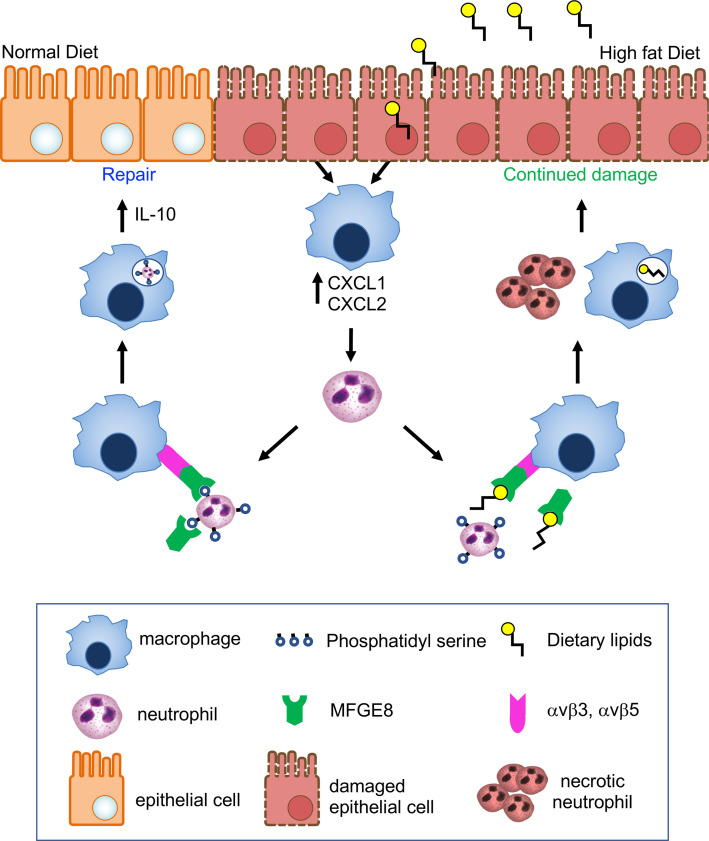

## Introduction

Within the intestine, a single layer of epithelial cells separates internal tissues from luminal contents. This forms a selective barrier, allowing for nutrient absorption while excluding harmful substances and intestinal microbes. Injury to this barrier increases entry of luminal contents into the tissue. Under normal conditions, microbial entry into tissue after damage is sensed by intestinal cells, including macrophages, which recruit neutrophils and other immune cells to clear infection and orchestrates tissue repair (1, 2).

Macrophages and epithelial cells secrete chemokines, such as CXCL1 and CXCL2, to recruit CXCR2-expressing neutrophils and support microbial clearance from the tissue (3–5). After microbial clearance, neutrophil effector mechanisms must be rapidly contained to prevent collateral tissue damage (6). Neutrophil apoptosis and subsequent phagocytosis by macrophages activate key signals for tissue healing and for dampening inflammation (6, 7).

A functional switch of macrophages from an antimicrobial, proinflammatory response to a pro–tissue repair, antiinflammatory response promotes barrier repair (6, 7). This includes macrophage production of antiinflammatory prorepair cytokine IL-10, which supports repair of intestinal damage (8–11). One inducer of this switch is macrophage phagocytosis of dead neutrophils (6, 12, 13). Defects in macrophage dead cell clearance results in impaired prorepair responses and lost IL-10 production and is linked to chronic inflammatory diseases such as atherosclerosis, cardiovascular disease, chronic obstructive pulmonary disease (COPD), and autoimmune diseases, including type 1 diabetes. Furthermore, enhanced or unresolved intestinal barrier damage underlies disease pathology in inflammatory bowel disease (IBD), including loss of IL-10 and increased tissue neutrophil accumulation (5, 14). Identifying mechanisms that support repair and understanding how these pathways are dysregulated in disease are key to improving resolution of tissue damage in IBD.

The intestinal tract is exposed to a variety of environmental factors with the potential to disrupt tissue repair and enhance disease pathology. One such factor is dietary lipids, with increased fat intake as a risk factor for chronic inflammatory diseases, including IBD (15). Chronic exposure to a high-fat diet (HFD) suppresses fat-associated macrophage antiinflammatory functions, including IL-10 production, and promotes proinflammatory responses, supporting local adipose tissue expansion and systemic inflammation (16–18). HFD increases susceptibility to intestinal damage and inflammation in colitis models (19–22). However, whether dysregulated repair mechanisms in early stages of HFD exposure sensitizes to the development or progression of intestinal disease is unclear.

HFD-induced altered macrophage functions is driven by macrophage lipid uptake (16–18). Studies suggest that dead cells and lipids are taken up through a shared pathway involving bridging molecule milk fat globule-EGF factor 8 (MFGE8). The discoidin domains of MFGE8 bind externalized phosphatidyl serine (PS) on the membrane of dead cells and induces uptake through macrophage αVβ3 (7, 23, 24). Similarly, the MFGE8 discoidin domains also bind dietary lipids, and this binding induces lipid uptake into adipocytes and promotes HFD-induced obesity (25). Disruption of MFGE8 binding to αVβ3 or loss of MFGE8 impairs apoptotic neutrophil and lipid uptake, highlighting the importance of this pathway in uptake of both and suggesting that this pathway is important in HFD regulation of macrophage function (24, 25).

In this study, we sought to determine the impact of short-term HFD feeding in intestinal injury. Using in vivo and in vitro studies, we demonstrate that HFD impairs resolution after intestinal injury by decreasing macrophage clearance of apoptotic neutrophils. We find this effect is driven by the direct interference of dietary lipids on MFGE8-mediated macrophage uptake of apoptotic neutrophils and subsequent IL-10 production.

These findings demonstrate a previously unidentified mechanism by which dietary lipids, a risk factor for intestinal disease, directly interfere with homeostatic processes required to resolve tissue injury. The breakdown of intestinal tissue repair responses in the early stages of HFD feeding may set the stage for enhanced inflammation seen after chronic HFD exposure.

## Results

### Acute HFD feeding impairs intestinal epithelial repair after injury.

**Figure 1:**
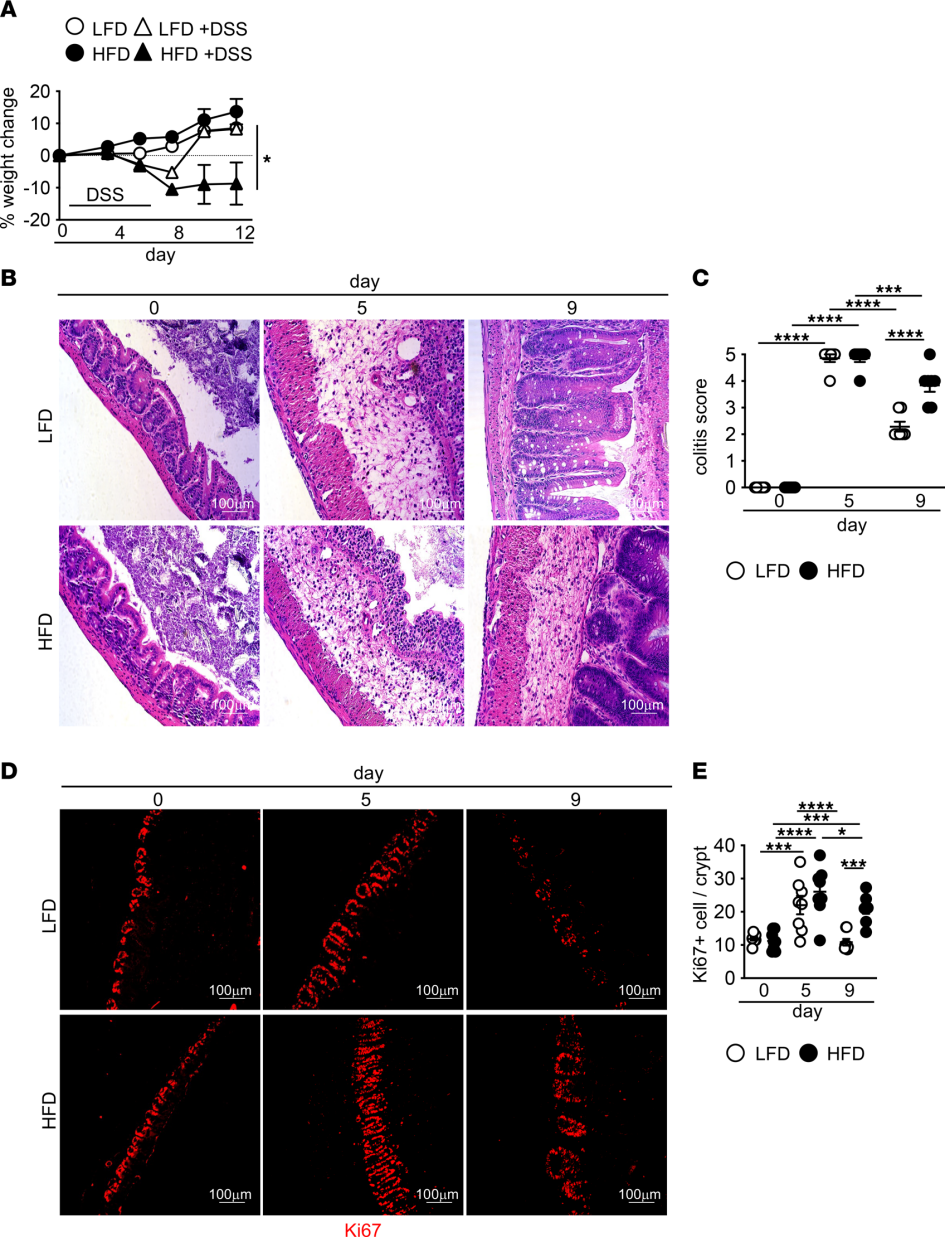
Impaired resolution of intestinal damage after acute high-fat diet. C57BL/6J mice were fed LFD or HFD for 1 week. Mice were then left untreated or treated with 2% DSS in drinking water for 5 days. (**A**) Body weight (*n* = 8 mice/group). The following panels are measurements in cecum of mice in **A**. (**B** and **C**) Representative H&E staining and blinded colitis score at indicated day after DSS treatment (*n* = 8 mice/group). (**D** and **E**) Representative image and quantification of proliferating cells (Ki67^+^) at indicated day after DSS treatment (*n* = 8 mice/group). An average of 3 high-powered filed (HPF) images per mouse was used for image quantification. Scale bar: 100 μm. Data are presented as mean ± SEM. *P < 0.05, ***P < 0.001, ****P < 0.0001. Statistical comparisons were performed using Student’s *t* test (**A**) and 1-way ANOVA with Tukey’s post hoc test (**C** and **E**), and if not indicated, a comparison is not significant.

In intestinal disease, long-term exposure to HFD is associated with increased pathology. However, whether the initial effects of HFD on damage repair sets the stage for increased pathology seen after chronic HFD feeding is unclear. To understand whether short-term exposure to HFD altered resolution after intestinal damage, we fed mice with a HFD or low-fat diet (LFD) for 1 week before exposure to dextran sodium sulfate (DSS), which causes intestinal epithelial damage in the cecum and colon (26, 27). Mice remained on their respective diets before, during, and after DSS treatment. We chose this timeframe in order to allow us to assess the impact of HFD on intestinal damage prior to the induction of metabolic and systemic inflammatory effects caused by chronic HFD feeding (28). Without DSS exposure, mice in both groups had comparable body weight with normal glucose and insulin tolerance, as compared with mice fed HFD for 8 weeks who had decreased glucose but normal insulin tolerance (Figure 1A and Supplemental Figure 1, A and B; supplemental material available online with this article; https://doi.org/10.1172/jci.insight.164489DS1). After DSS treatment, mice exposed to HFD or LFD initially lost a comparable amount of weight. While LFD-fed mice recovered initial weight, HFD-fed mice showed sustained weight loss (Figure 1A). By histopathological analysis, we found that acute HFD feeding alone did not increase tissue damage or inflammation in the cecum, terminal ileum, or colon (Figure 1, B and C, and Supplemental Figure 1, C–E). At day 5 of DSS treatment, we found increased pathology in the cecum, terminal ileum, proximal, and distal colon with equivalent colitis scores in both HFD- and LFD-treated animals (Figure 1, B and C, and Supplemental Figure 1, C–E), indicating that diet was not amplifying initial damage or inflammation, as is seen after chronic exposure to HFD (22, 29, 30). In LFD- and HFD-fed mice, by 4 days after DSS treatment (day 9), the damage was partially resolved in the terminal ileum and proximal and distal colon (Supplemental Figure 1, C–E) with reduced tissue infiltration of immune cells and restitution of the epithelial barrier. In the cecum of LFD-fed mice, we observed a similar resolution of damage (Figure 1, B and C). In contrast, in HFD-fed mice, we observed prolonged tissue damage and cellular infiltration (Figure 1, B and C).

HFD consumption is associated with microbiota compositional changes, which are suggested to be a mechanism for HFD-driven intestinal inflammation (31). Changes in microbiota composition with outgrowth of specific taxa are also associated with increased inflammation and can support pathology after DSS treatment (32–34). Increased damage to intestinal tissue can also cause shifts in intestinal microbe composition (33). To determine if microbiota changes occurred after acute HFD and DSS treatment and whether these changes could account for sustained cecal damage, we performed 16S rRNA-Seq to assess cecal microbial composition. At the phylum level (Supplemental Figure 1F), only Proteobacteria and Actinobacteria were increased in the cecum of HFD-fed mice before DSS treatment, with similar levels between diet groups after DSS. Firmicutes decreased in both groups after DSS treatment, with a greater decrease in HFD-fed mice. Bacteroidetes increased similarly in both groups after DSS treatment. There were minimal changes at the family level. In HFD-fed mice, *Desulfovibrionaceae*, *Clostridiales*, and *Lachnospiraceae* were increased before DSS treatment. After DSS treatment, *Desulfovibrionaceae*, *Clostridiales*, and *Lachnospiraceae* levels were equivalent between diet groups (Supplemental Figure 1G). After DSS treatment, we found increased Bacteroidaceae in HFD-treated mice (Supplemental Figure 1G). In LFD-treated mice, we found increased *Erysipelotrichaceae* that deceased after DSS treatment but was still elevated as compared with HFD-fed mice (Supplemental Figure 1H). After DSS treatment, *Bacteroidales* and *Porphyromonadaceae* increased in the cecum of LFD-fed mice (Supplemental Figure 1H). *Sutterellaceae* was elevated in both diet groups after DSS treatment (Supplemental Figure 1I). Increased levels of *Erysipelotrichaceae*, *Bacteroidales*, and *Porphyromonadaceae* are associated with enhanced disease in patients with IBD and mouse models of intestinal inflammation (35–37), while Bacteroidaceae, is reduced in patients with IBD (38). To confirm that altered microbes were not sufficient to impede repair after DSS treatment, we transplanted cecal microbiota contents from LFD- and HFD-fed mice into antibiotic-treated mice, which we then treated with DSS. Colonization of mice with cecal contents from HFD-treated mice did not phenocopy HFD feeding itself, as mice did not have sustained weight loss as compared with mice colonized with cecal microbes from LFD-fed mice (Supplemental Figure 1J). Since we did not find expansion of microbes associated with enhanced intestinal pathology after HFD feeding or sustained disease after transplant of cecal microbes from HFD-fed mice, we do not think HFD-driven changes in microbial composition underlies lack of resolution of cecal damage after short-term HFD feeding.

To understand why mice on HFD had impaired damage resolution only in the cecum, we asked if lipids specifically accumulated in the cecum. To assess lipid content, we used BODIPY, which stains neutral lipids. BODIPY staining intensity was similar between the cecum and colon of LFD-fed mice (Supplemental Figure 1K). In HFD-fed mice, there was increased BODIPY staining in the cecum, as compared with the colon. When comparing between diet groups, HFD-fed mice has increased lipids in the cecum but not colon, as compared with LFD-fed mice (Supplemental Figure 1K), suggesting that increased cecal lipids are associated with lack of tissue repair. Based on these findings, we focused the remainder of our study on the cecum.

One of the first steps in repairing intestinal epithelial damage is proliferation of intestinal stem cells (ISC) followed by differentiation to repopulate lost cell types (39). Chronic HFD exposure alone can alter colonic ISC proliferation, resulting in increased ISC numbers (40). We next assessed if ISC proliferation in response to damage was different in LFD- versus HFD-fed mice. Before DSS and at day 5 of DSS treatment, we found similar epithelial proliferation in the cecum of HFD- and LFD-fed mice (Figure 1, D and E). By day 9, proliferation in the cecum decreased to pre-DSS levels in the LFD-treated mice. However, it remained elevated in the HFD-treated mice (Figure 1, D and E). These findings demonstrate that sustained ISC proliferation maintained increased numbers of ISC in cecal crypts but did not support the reestablishment of the epithelial barrier (Figure 1, B and C).

**Figure 2:**
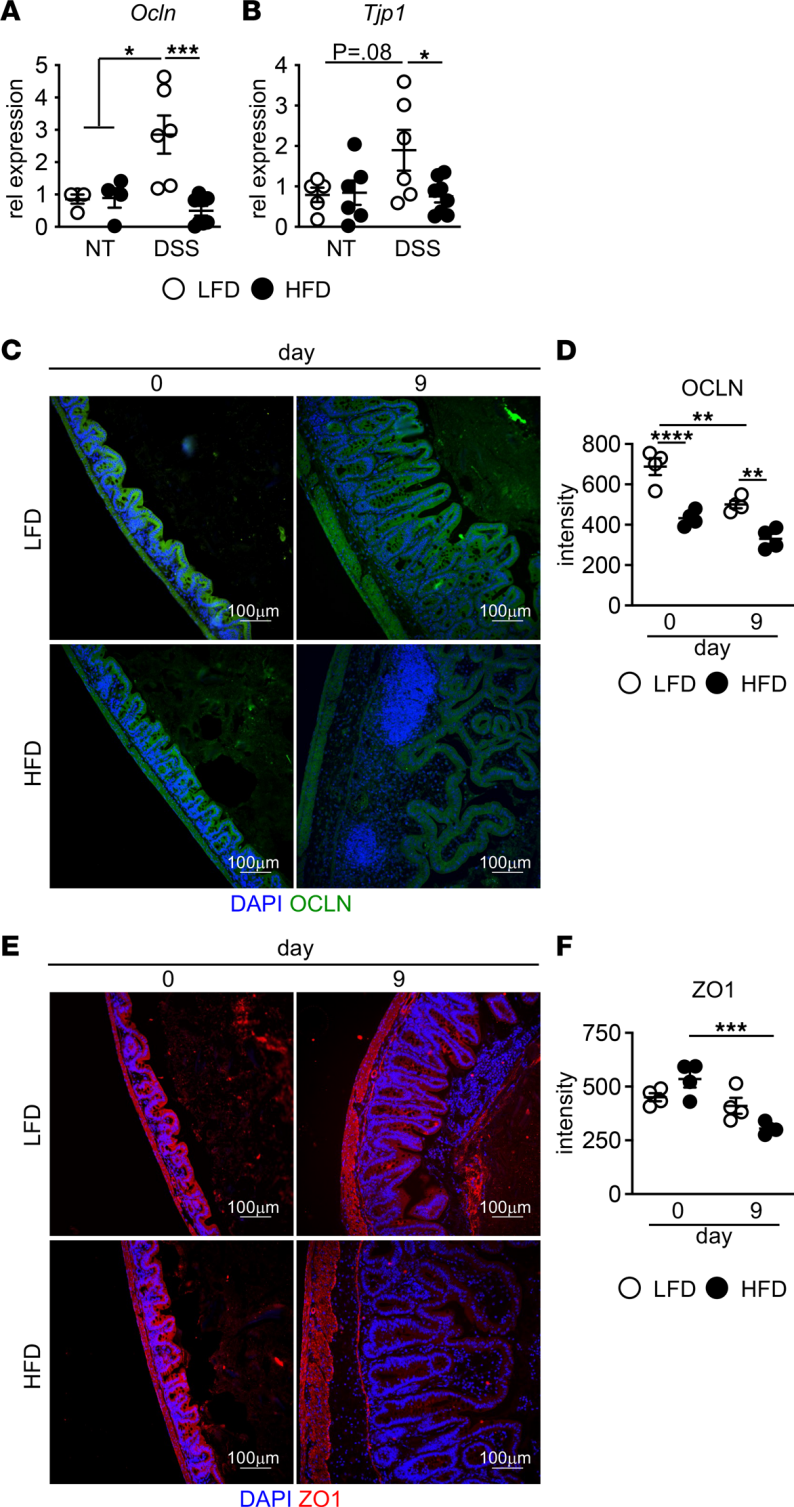
Decreased tight junctions in cecum of HFD-fed mice after DSS. (**A** and **B**) Occludin (*Ocln*) (*n* = 4 LFD and HFD, *n* = 6 LFD DSS, *n* = 8 HFD DSS) and Zo1 (Tjp1) gene expression at day 7 days after DSS treatment (*n* = 6 LFD, HFD, LFD DSS; *n* = 8 HFD DSS). (**C**–**F**) Representative image and quantification of Occludin (OCLN) (**C** and **D**) and ZO1 (**E** and **F**) at indicated day after DSS treatment (*n* = 4 mice/group). An average of 3 high-powered filed (HPF) images per mouse was used for image quantification. Scale bar: 100 μm. Data are presented as mean ± SEM. *P < 0.05, **P < 0.01, ***P < 0.001, ****P < 0.0001. Statistical comparisons were performed using Student’s *t* test (**A** and **B**) and 1-way ANOVA with Tukey’s post hoc test (**D** and **F**), and if not indicated, a comparison is not significant.

In order to form a tight seal between repopulated epithelial cells in repair of intestinal damage, tight junctions must be reestablished. This paracellular barrier prevents microbial translocation into the tissue (41). Expression of tight junction proteins occludin (Ocln) and zonula occluden-1 (ZO1) — which, together, are the major tight-junction proteins (42) — did not differ in the cecum between LFD- and HFD-fed mice before DSS treatment (Figure 2, A and B). By day 7, we found upregulated expression of Ocln and Tjp1 in the cecum of LFD, but not HFD treated mice (Figure 2, A and B). Next, we performed immunofluorescence (IF) staining to determine differences in OCLN and ZO1 cecal protein expression in the epithelium. OCLN, but not ZO1, protein was decreased in the epithelium of HFD-fed mice at day 0 prior to DSS treatment (Figure 2, C–F). Similar effects on OCLN expression in the distal ileum adjacent to the cecum have been seen in long-term HFD feeding (20). Previous studies demonstrate that OCLN and ZO1 expression decreases after DSS treatment (43). By day 9 after DSS treatment, OCLN was reduced in both diet groups, with lower expression in HFD-fed mice as compared with LFD-fed mice (Figure 2, C and D). ZO1 expression in LFD-fed mice at day 9 after DSS was comparable with mice before DSS treatment. In contrast, ZO1 expression was reduced in HFD-fed mice after DSS treatment (Figure 2, E and F).

**Figure 3:**
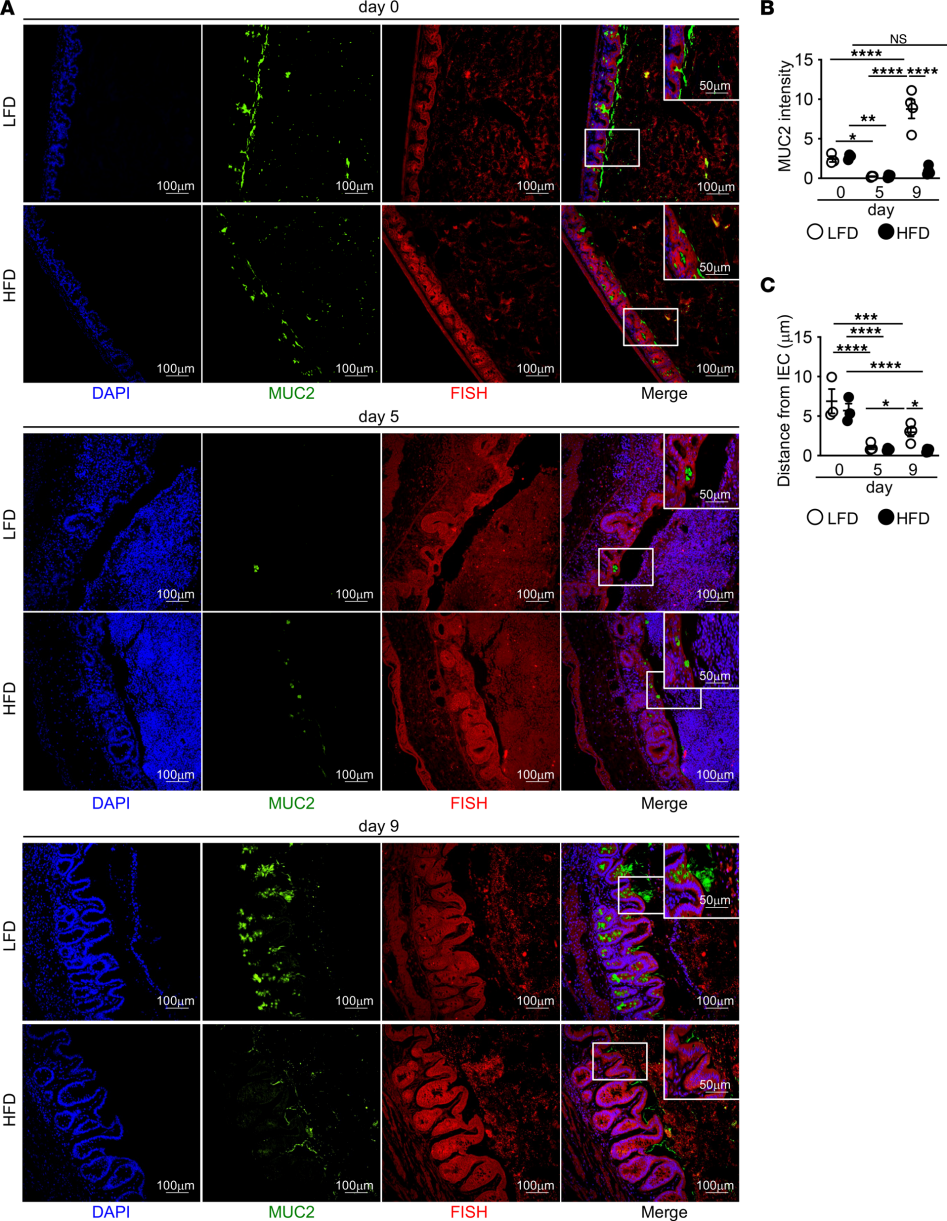
Decreased mucus production and increased epithelial bacterial encroachment in cecum of HFD-fed mice after DSS. (**A**–**C**) Immunofluorescence staining for mucus (MUC2, green) and bacteria (FISH, red) and quantification of MUC2 and bacterial distance from IEC in LFD and HFD mice at indicated day of DSS treatment (*n* = 3 day 0, *n* = 4 day 5 and day 9 mice/group). An average measurement of 3 high-powered filed (HPF) images per mouse was used for image quantification. Scale bar: 100 μm or 50 μm, as indicated. Data are presented as mean ± SEM. *P < 0.05, **P < 0.01, ***P < 0.001, ****P < 0.0001. Statistical comparisons were performed using 1-way ANOVA with Tukey’s post hoc test (**B** and **C**), and if not indicated, a comparison is not significant.

Restoration of the intestinal barrier includes the repopulation of goblet cells and mucus, which aid in decreasing inflammatory microbial-epithelial interactions (44). We performed Alcian blue/PAS (ABPAS) staining to assess goblet cell numbers and found decreased goblet cells at day 9 after DSS in HFD-fed mice as compared with LFD-fed mice (Supplemental Figure 1L). We next performed FISH for bacteria and MUC2 staining for mucus in the cecum of LFD- and HFD-fed mice before and after DSS treatment to assess these interactions. The mucus barrier in the cecum was equivalent in both diet groups prior to DSS treatment and was similarly disrupted at day 5 of DSS, with extensive interactions between intestinal microbes and the cecal epithelium in both groups (Figure 3, A–C). While mucus production was elevated in LFD-treated mice by day 9, mucus production remained low in the cecum of HFD-treated mice (Figure 3, A and B). While there was good separation by day 9 of microbes from the epithelium in the LFD-treated mice, there was little separation between microbes and epithelial cells in the cecum of HFD-treated mice (Figure 3, A and C). These increased interactions with intestinal microbes likely support sustained tissue damage and inflammation in HFD-fed mice.

### Continued neutrophil accumulation limits damage repair after DSS in HFD-treated mice.

**Figure 4:**
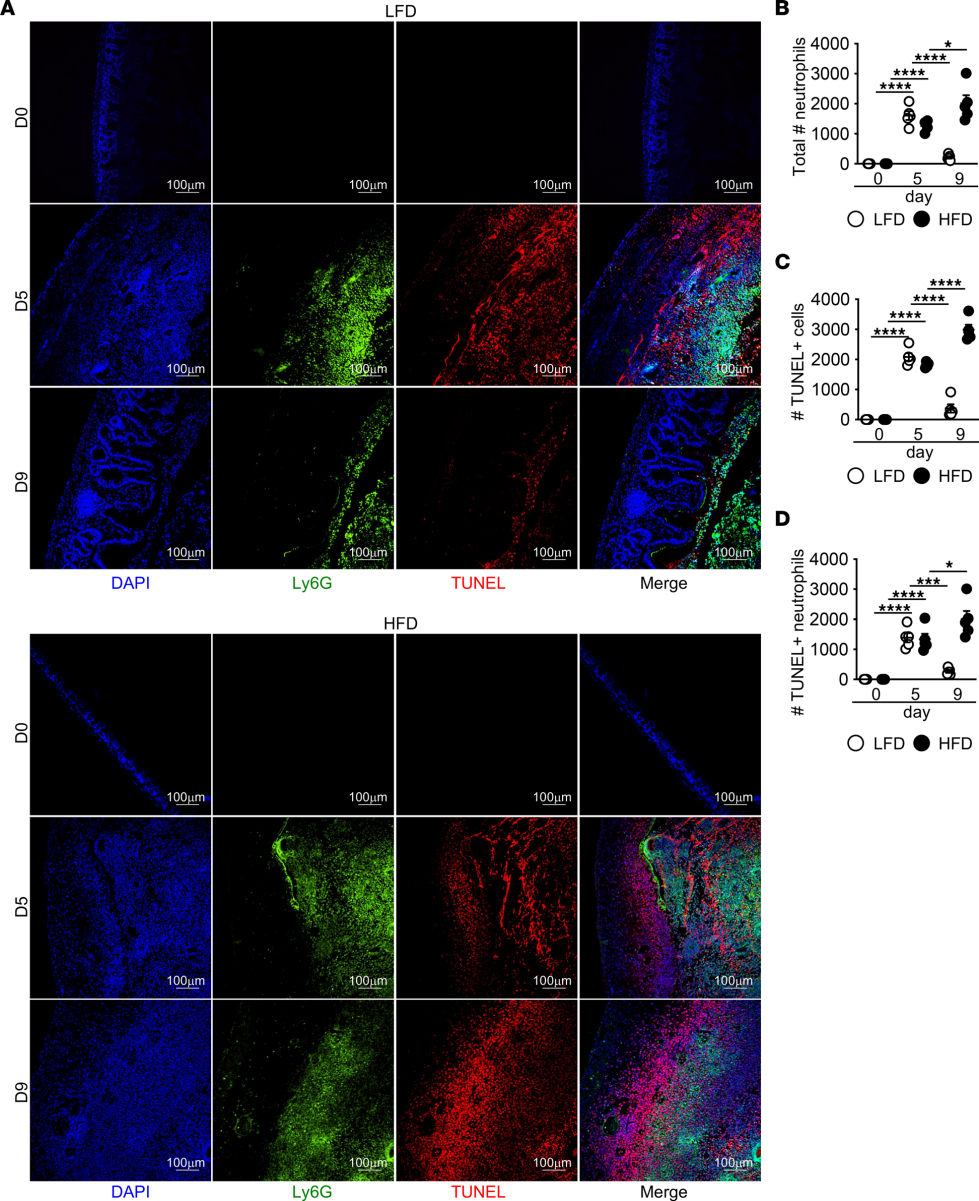
Continued dead neutrophil accumulation in cecum of HFD-fed mice after DSS. (**A**–**D**) Representative staining and quantification of total neutrophils (Ly6G^+^), total TUNEL^+^ cells, and TUNEL^+^ neutrophils in cecum of LFD and HFD control and DSS mice (*n* = 4 day 0, *n* = 5 day 5 and day 9 mice/group). An average measurement of 3 high-powered filed (HPF) images per mouse was used for image quantification. Scale bar: 100 μm. Data are presented as mean ± SEM. *P < 0.05,***P < 0.001, ****P < 0.0001. Statistical comparisons were performed using 1-way ANOVA with Tukey’s post hoc test, and if not indicated, a comparison is not significant.

During intestinal damage, interactions of luminal microbes with the epithelium and translocation into damaged tissue induces neutrophil recruitment into both the lamina propria and intestinal lumen (5), enabling microbial clearance. However, continued recruitment and increased neutrophil numbers can be pathological to the tissue (45–47). Neutrophils are short-lived cells that die by apoptosis to prevent the release of cytotoxic intracellular components (48). One mechanism that aids in induction of tissue repair is the clearance of dead neutrophils from the tissue to prevent secondary necrosis, which amplifies tissue pathology and limits intestinal barrier repair (5, 7). Defects in apoptotic neutrophil clearance is associated with impaired tissue repair (6). By H&E staining, we observed neutrophils in the cecum and lumen of both LFD- and HFD-fed mice after DSS treatment, and it remained elevated in HFD-fed mice (Supplemental Figure 2A). Since dead neutrophil accumulation can amplify tissue damage, we next costained for apoptotic cells using TUNEL and neutrophil marker Ly6G to assess neutrophil numbers and viability in the tissue. Prior to DSS treatment, we found no TUNEL or Ly6G staining in the cecum of either group (Figure 4, A–C). We saw similarly increased numbers of neutrophils and dead cells (the majority of which were neutrophils) in the cecum of HFD- and LFD-fed mice at day 5 of DSS (Figure 4, A–D). At day 9, while neutrophils, dead cells, and dead neutrophil numbers decreased in the cecum of LFD-treated mice, they further increased in the cecum of HFD-treated mice (Figure 4, A–D).

**Figure 5:**
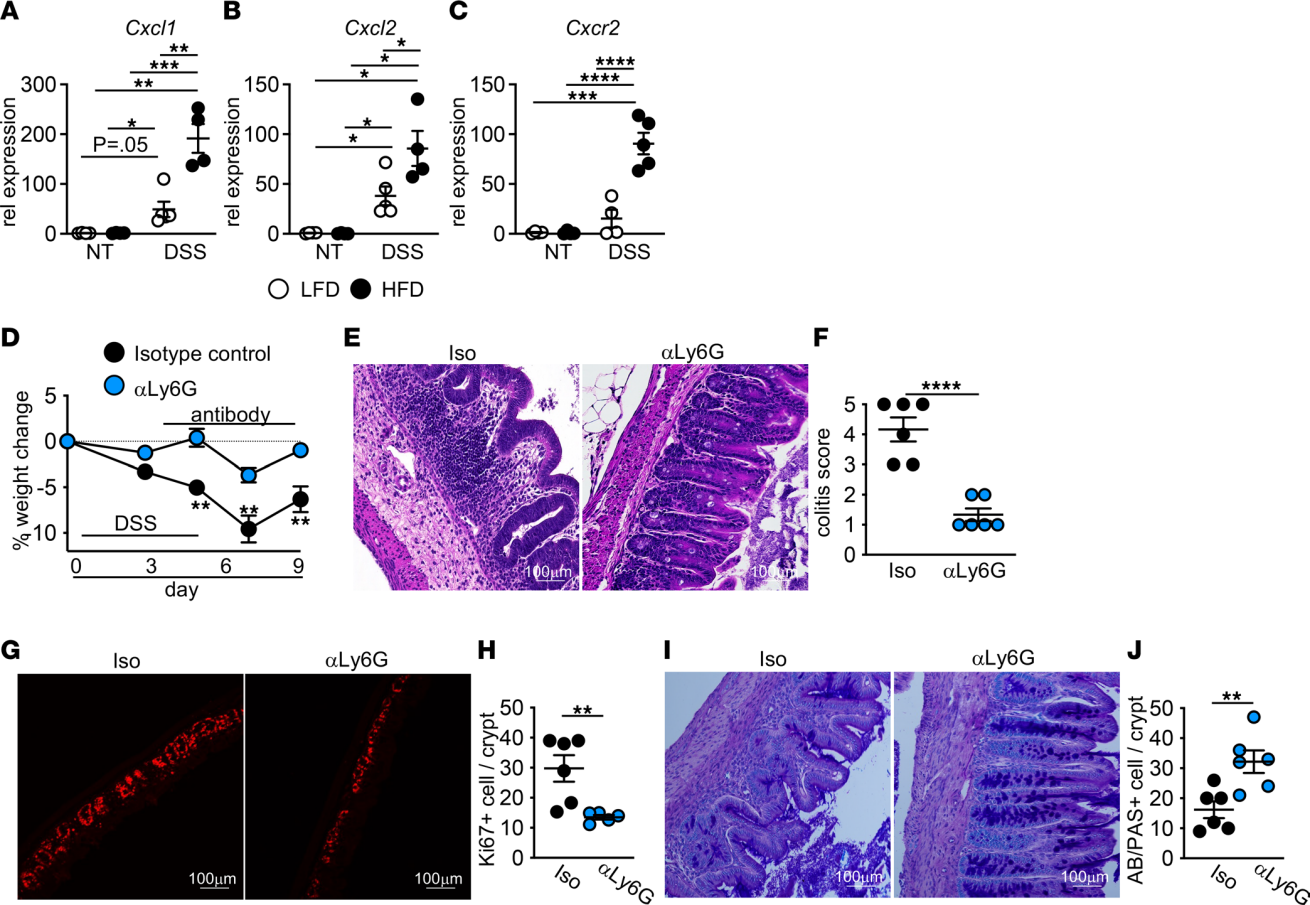
Neutrophil depletion restores damage resolution in HFD-fed mice. (**A**–**C**) *Cxcl1*, *Cxcl2*, and *Cxcr2* gene expression in cecum of LFD- and HFD-fed control and DSS-treated mice (*n* = 4 mice/group). Data shown are representative of 2 experiments. (**D**–**J**) At day 4 of DSS, HFD-fed mice received a single dose of anti-IgG2a isotype control or anti-Ly6g (*n* = 6 mice/group). (**D**) Body weight changes. (**E**) Representative H&E images. (**F**) Blinded colitis score. (**G**) Representative staining. (**H**) Quantification for proliferating cells. (**I**) Representative Alcian blue/PAS staining. (**J**) Quantification of goblet cells. An average measurement of 3 high-powered filed (HPF) images per mouse was used for image quantification. Data are presented as mean ± SEM. *P < 0.05, **P < 0.01, ***P < 0.001, ****P < 0.0001. Statistical comparisons were performed using 1-way ANOVA with Tukey’s post hoc test (**A**–**C**) or Student’s *t* test (**D**, **F**, **H**, and **J**), and if not indicated, a comparison is not significant. Scale bar: 100 μm.

In inflammation resolution, neutrophil-recruiting chemokine expression is reduced. We examined gene expression of neutrophil chemoattractant proteins CXCL1 and CXCL2 and chemokine receptor CXCR2 in LFD- and HFD-fed mice before and after DSS treatment. At day 7 after DSS treatment, expression of *Cxcl1*, *Cxcl2*, and *Cxcr2* were increased in the cecum of both groups and were further elevated in HFD-fed mice (Figure 5, A–C). Continued recruitment and accumulation of dead neutrophils correlated with the enhanced pathology we observe in the cecum of HFD-fed DSS-treated mice.

**Figure 6:**
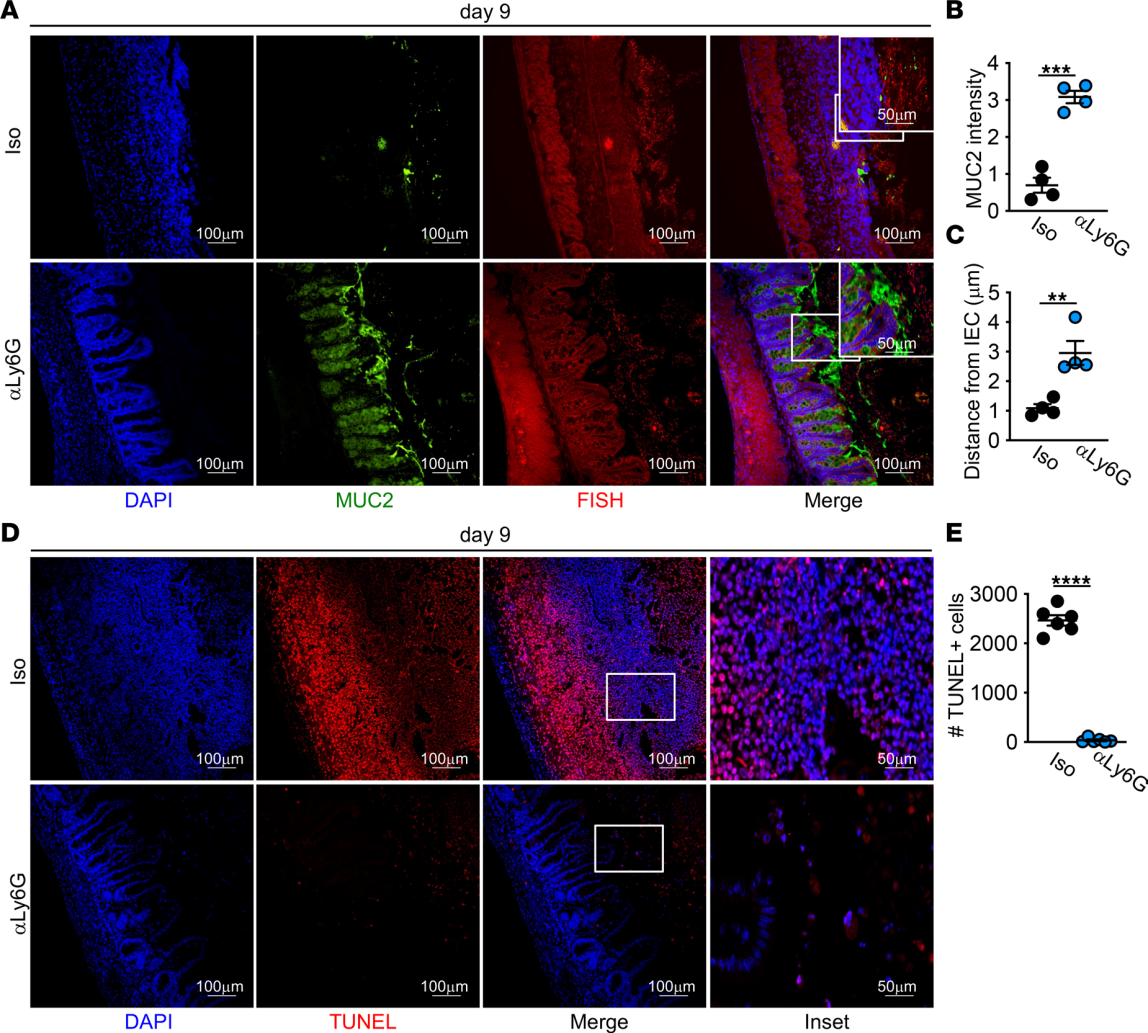
Neutrophil depletion restores mucus production with decreased epithelial bacterial encroachment in cecum of HFD-fed mice after DSS. (**A**–**C**) Representative image for nuclei, mucus, and bacteria, and quantification of MUC2 intensity and bacterial encroachment (*n* = 4 mice/group). (**D** and **E**) Representative staining and quantification of TUNEL^+^ cells. An average measurement of 3 high-powered filed (HPF) images per mouse was used for image quantification. Data are presented as mean ± SEM. **P < 0.01, ***P < 0.001,****P < 0.0001. Statistical comparisons were performed using Student’s *t* test, and if not indicated, a comparison is not significant. Scale bar: 100 μm or 50 μm, as indicated.

To investigate whether neutrophils contributed to reduced tissue repair after acute HFD feeding, we depleted neutrophils in HFD-fed mice starting at day 4 of DSS treatment after the induction of damage. As compared with isotype control antibody–treated mice, neutrophil depletion resulted in improved body weight, improved histopathology, decreased epithelial proliferation, and restored goblet cell numbers (Figure 5, D–J). Neutrophil depletion resulted in the restoration of the mucus barrier and reduced mucosal-associated microbes (Figure 6, A–C). Depletion of neutrophils also resulted in decreased numbers of TUNEL^+^ cells within the tissue (Figure 6, D and E). Collectively, these data suggest that continued neutrophil recruitment and accumulation of dead neutrophils supports defective resolution after injury in HFD-fed mice.

### Acute HFD feeding impairs intestinal macrophage clearance of dead neutrophils.

Macrophage recruitment into the tissue is key to recovery from injury, including clearance of apoptotic cells (49, 50). We first tested if normal numbers of macrophages were recruited into the tissue. By IF staining and flow cytometric analysis, we confirmed equivalent macrophage recruitment into the tissue after DSS treatment in both HFD- and LFD-fed mice (Supplemental Figure 2, B–G).

**Figure 7:**
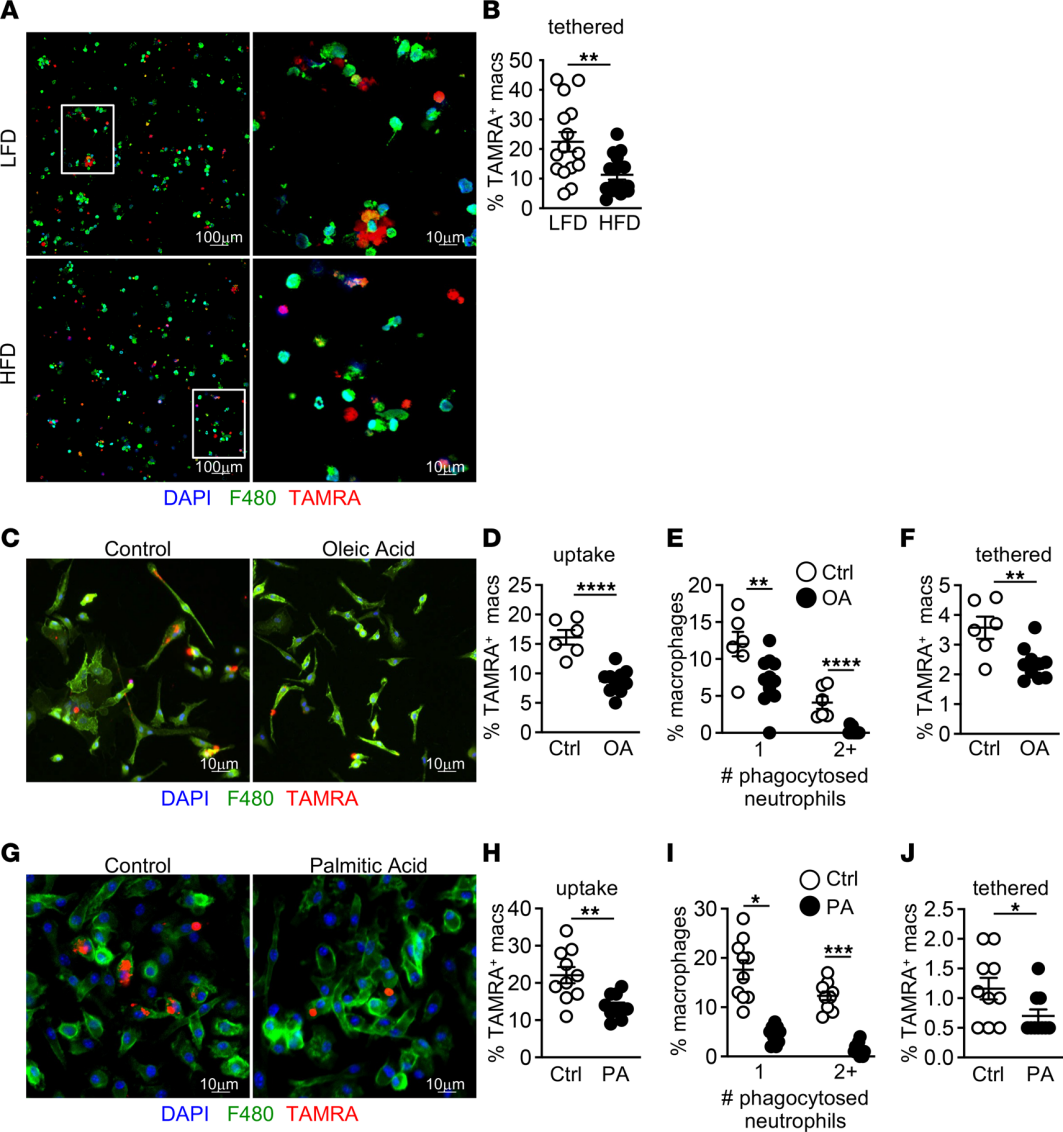
Lipids impair macrophage uptake of dead neutrophils. (**A** and **B**) Representative image and quantification of the percentage of tethered TAMRA^+^ neutrophils in sorted macrophages from LFD- and HFD-fed mice after DSS treatment. Data shown are representative of 2 experiments (*n* = 11 LFD DSS and *n* = 15 HFD DSS HPF images). (**C** and **D**) Representative immunofluorescence image and quantification from phagocytosis assay stained for macrophages, nuclei, and dead neutrophils (TAMRA^+^) in control (Ctrl) or oleic acid (OA) pretreated BMDMs. The percentage of TAMRA^+^ macrophages is indicative of the percentage of macrophages that phagocytosed TAMRA^+^ dead neutrophils. (**E**) Quantification of the percentage of BMDMs that have phagocytosed 1 or 2 or more TAMRA^+^ neutrophils after Ctrl or OA pretreatment. (**F**) Quantification of percent of BMDMs with tethered TAMRA^+^ neutrophils after Ctrl or OA pretreatment. Data shown are representative of 2 experiments (*n* = 6 control and *n* = 10 oleic acid HPF images). (**G**–**J**) Similar measurements as in **B**–**D** in Ctrl or palmitic acid (PA) pretreated BMDMs. Data shown are representative of 2 experiments (*n* = 10 control and palmitic acid HPF images). Data are presented as mean ± SEM. *P < 0.05, **P < 0.01, ***P < 0.001, ****P < 0.0001. Statistical comparisons were performed using Student’s *t* test, and if not indicated, a comparison is not significant. Scale bar: 100 μm or 10 μm, as indicated.

Macrophage clearance of apoptotic neutrophils is one mechanism that supports antiinflammatory signaling and tissue repair (6, 7). To identify whether tethering of dead neutrophils, the first step of neutrophil uptake, differed in macrophages from LFD- and HFD-fed mice, we exposed flow-sorted intestinal macrophages from DSS-treated HFD- and LFD-fed mice to TAMRA-labeled dead neutrophils and measured tethering by fluorescence microscopy; we saw reduced tethering of dead neutrophils by macrophages from HFD-treated mice (Figure 7, A and B).

We hypothesized that lipid components from the HFD were directly interfering with macrophage uptake of dead neutrophils. The major lipids in the HFD are oleic acid (50%) and palmitic acid (49%; Nu-Chek Prep, N-16-A). To test if these lipids interfered with macrophage uptake of neutrophils, we treated bone marrow–derived macrophages (BMDMs) with oleic acid before incubation with TAMRA-labeled dead neutrophils and assessed neutrophil engulfment by fluorescence microscopy. After pretreatment with oleic acid, we found that BMDMs had a decreased capacity to engulf dead neutrophils with a decreased proportion containing a single neutrophil and none containing more than 1 cell (Figure 7, C–E). In order to engulf dead cells, macrophages must first tether to the exposed phosphatidylserine (PS) on the dead cell membrane. Next, to assess macrophage tethering of dead neutrophils, we performed our uptake assays at 4°C to decrease phagocytosis. While control-treated BMDMs tethered dead neutrophils, oleic acid treatment decreased the ability of BMDMs to tether dead neutrophils (Figure 7F). We found similar results when BMDMs were exposed to palmitic acid, with decreased uptake and tethering (Figure 7, G–J). Using immortalized BMDMs (iBMDMs), we found that this effect was limited to dead neutrophils, as uptake of beads or dead thymocytes was not impacted by oleic acid treatment (Supplemental Figure 2, H–K). These findings demonstrate that lipid components of the HFD directly impair macrophage uptake of dead neutrophils and likely contribute to apoptotic neutrophil accumulation in HFD-fed mice after injury.

### Acute HFD treatment limits macrophage IL-10 production after intestinal injury.

**Figure 8:**
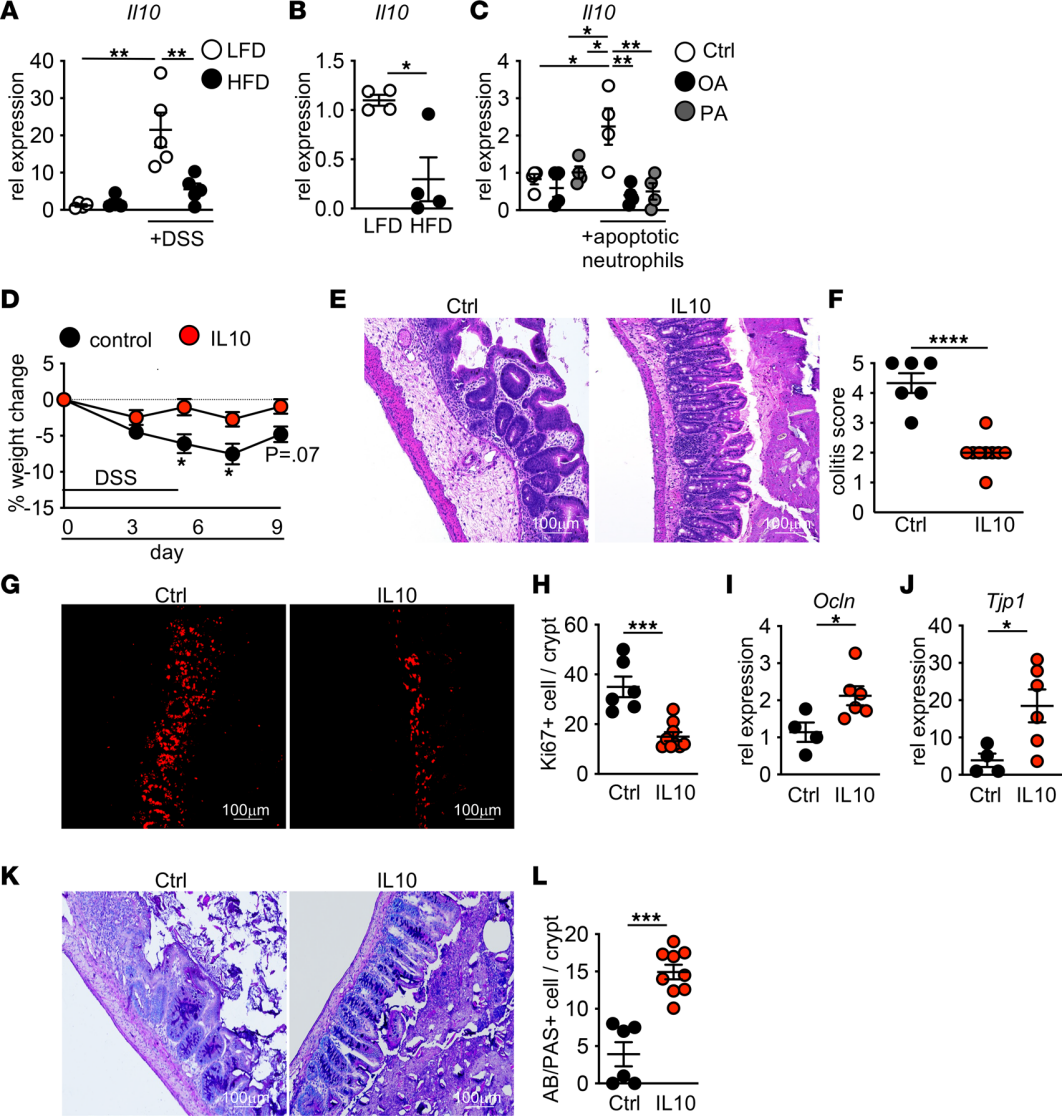
Lost Il10 expression after HFD feeding and DSS treatment. (**A** and **B**) *Il10* gene expression from cecum (*n* = 4 LFD and HFD, *n* = 5 LFD DSS and HFD DSS) (**A**) and intestinal (**B**) CX_3_CR1^+^ macrophages from LFD- and HFD-fed DSS-treated mice (*n* = 4 samples/group). (**C**) *Il10* gene expression in control (Ctrl), oleic acid (OA), or palmitic acid (PA) pretreated macrophages after exposure to dead neutrophils (2 pooled experiment with *n* = 4 technical replicates/group). (**D**) Body weight change in HFD-fed DSS-treated mice after hydrodynamic delivery of control or IL-10–producing plasmid (*n* = 6 control and *n* = 9 IL-10 mice/group). (**E**–**L**) measurements in cecum of mice in **D** (*n* = 6 mice per group). (**E** and **F**) Representative H&E staining and blinded colitis score. (**G** and **H**) Representative staining and quantification of Ki67^+^ proliferating cells. (**I** and **J**) Occludin (Ocln) and Zo1 (Tjp1) expression. (**K** and **L**) Representative Alcian blue/PAS staining and quantification of goblet cells. For all imaging, the average of 3 HPF images was taken per mouse. Data are presented as mean ± SEM. *P < 0.05, **P < 0.01,***P < 0.001, ****P < 0.0001. Statistical comparisons were performed using Student’s *t* test (**B**, **D**, **F**, **H–J**, and **L**) and 1-way ANOVA with Tukey’s post hoc test (**A** and **C**), and if not indicated, a comparison is not significant. Scale bar: 100 μm.

Macrophage clearance of apoptotic neutrophils regulates pathways important for barrier repair. This includes decreased expression of neutrophil chemoattractant proteins (Figure 5, A–C) (7). Macrophage uptake of dead cells also induces expression of antiinflammatory and tissue repair factors, including the antiinflammatory cytokine IL-10 (12, 51). IL-10 supports epithelial barrier repair (8, 9), regulates T cell responses to the intestinal microbiota and pathogens (52), and increases macrophage phagocytic capacity. While we found *Il10* gene expression upregulated in the cecum of LFD-fed mice after DSS treatment, *Il10* was not upregulated in the cecum of DSS-treated mice acutely exposed to HFD (Figure 8A). In the intestine, we and others find macrophages are the major source of IL-10 (8, 9, 52–54). After DSS exposure, *Il10* expression by cecal macrophages from mice on HFD was reduced as compared with macrophages from LFD-fed mice (Figure 8B). Total tissue and macrophage expression of proinflammatory genes such as *Tnf* were not altered after HFD treatment (Supplemental Figure 3, A and B). These findings demonstrate that *Il10* expression is lost in cecal macrophages in HFD-treated mice in response to tissue injury.

To determine if HFD lipids directly limited macrophage *Il10* expression in response to dead cells, we pretreated BMDMs with oleic or palmitic acid before exposure to dead neutrophils. Exposure to dead neutrophils increased *Il10* expression. However, *ll10* was not upregulated after lipid pretreatment (Figure 8C). Together, these findings demonstrate that dietary lipids can inhibit macrophage production of antiinflammatory signals needed to support intestinal tissue repair.

### IL-10 overexpression rescues tissue repair defects in HFD-fed mice.

**Figure 9:**
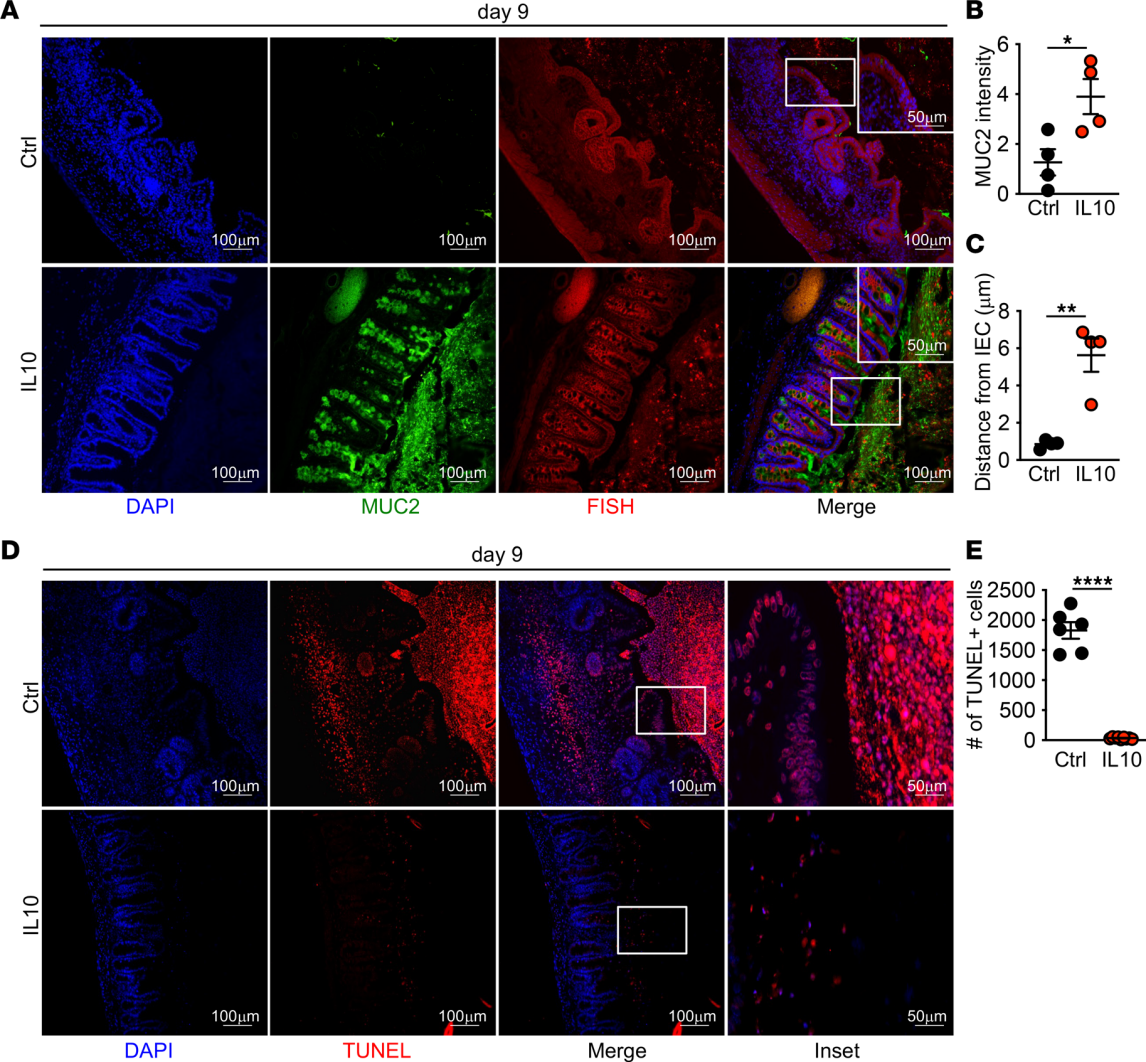
Il10 overexpression restores mucus production and decreases epithelial bacterial encroachment in cecum of HFD-fed mice after DSS. (**A**–**C**) Representative staining for nuclei, mucus, and bacteria and quantification of MUC2 intensity and bacterial encroachment (*n* = 4 mice/group). (**D** and **E**) Representative image and quantification of TUNEL^+^ cells (*n* = 6 mice per group). For all imaging, the average of 3 HPF images was taken per mouse. Data are presented as mean ± SEM. *P < 0.05, **P < 0.01, ****P < 0.0001. Statistical comparisons were performed using Student’s *t* test (**B**, **C**, and **E**), and if not indicated, a comparison is not significant. Scale bar: 100 μm or 50 μm, as indicated.

To understand if IL-10 expression was sufficient to protect mice from intestinal damage in the presence of HFD, we overexpressed IL-10 in vivo (Supplemental Figure 3C) and found it protected HFD-treated mice from increased weight loss after DSS treatment (Figure 8D). We also observed improved tissue histology, reduced epithelial proliferation, and increased expression of gap junction proteins (Figure 8, E–J). We also found restored goblet cell numbers (Figure 8, K and L). IL-10 overexpression restored mucus production with reduced microbial interactions with the epithelium (Figure 9, A–C) alongside reduced accumulation of dead cells (Figure 9, D and E). These findings demonstrate that, as seen under normal dietary conditions, IL-10 is sufficient to normalize barrier repair in HFD-treated mice after intestinal injury, suggesting that loss of this response in macrophages exposed to dead neutrophils contributes to defective cecal tissue repair in mice fed HFD.

The importance of IL-10 signaling in epithelial cells in the repair of intestinal damage has been shown by many other groups (8, 9). To determine whether IL-10 signaling induces repair in HFD-fed mice with intestinal injury, we overexpressed IL-10 in HFD-fed mice lacking IL-10 receptor α (IL-10Rα) on macrophages (Supplemental Figure 3D). IL-10Rα on macrophages is dispensable, as IL-10 overexpression rescued HFD-treated mice lacking macrophage IL-10Rα after DSS treatment (Supplemental Figure 3, E–I). We then overexpressed IL-10 in HFD-fed mice lacking the IL-10Rα on epithelial cells (Supplemental Figure 3J). In contrast, IL-10 overexpression did not rescue pathology in HFD-fed mice lacking IL-10Rα on epithelial cells with increased body weight loss and histopathology in the presence of exogenous IL-10 (Supplemental Figure 3, K and L). By histology, we observed very few crypt structures, indicating this signaling pathway may also be critical for stem cell renewal. Excess epithelial proliferation, loss of goblet cells, dead cell accumulation, and the mucus barrier were also not rescued by IL-10 overexpression in HFD DSS-treated mice where epithelial cells lack IL-10Rα (Supplemental Figure 3, M–P). These data demonstrate that IL-10 signaling on intestinal epithelial cells supports repair of cecal damage in HFD-fed mice.

### Dietary lipids interfere with MFGE8-mediated macrophage uptake of apoptotic neutrophils.

We sought to identify a potential mechanism of dietary lipid interference with macrophage clearance of apoptotic neutrophils. Previously described pathways for apoptotic neutrophil uptake into macrophages are identified as also important for cellular uptake of dietary lipids (25). The bridging molecule MFGE8 and its receptor αVβ3 facilitate apoptotic neutrophil uptake into macrophages (7, 23, 24). The MFGE8 discoidin domain binds to externalized PS on dead neutrophils and the Gly-Arg-Gly-Asp-Asn-Pro (RGD) motif of MFGE8 binds to αVβ3 (24). Recent studies show that dietary lipid uptake into adipocytes is facilitated by MFGE8 and αVβ3 with lipid binding to the discoidin domains of MFGE8 (25). Together, these studies demonstrate a shared mechanism of apoptotic neutrophil and lipid uptake.

Due to the dual role of αVβ3 and MFGE8 in PS and dietary lipid uptake into cells, we next asked whether αVβ3 and MFGE8 expression was altered in LFD- and HFD-fed mice in response to intestinal injury, as loss of MFGE8 or αVβ3 can impair apoptotic neutrophil and lipid uptake (23–25). By flow cytometry, we found similar surface expression of αVβ3 on cecal macrophages from LFD- and HFD-fed mice after DSS treatment (Supplemental Figure 4A). No change in expression was seen for other PS receptors, including *Axl* and *Mertk* (55) (Supplemental Figure 4, B–E).

**Figure 10:**
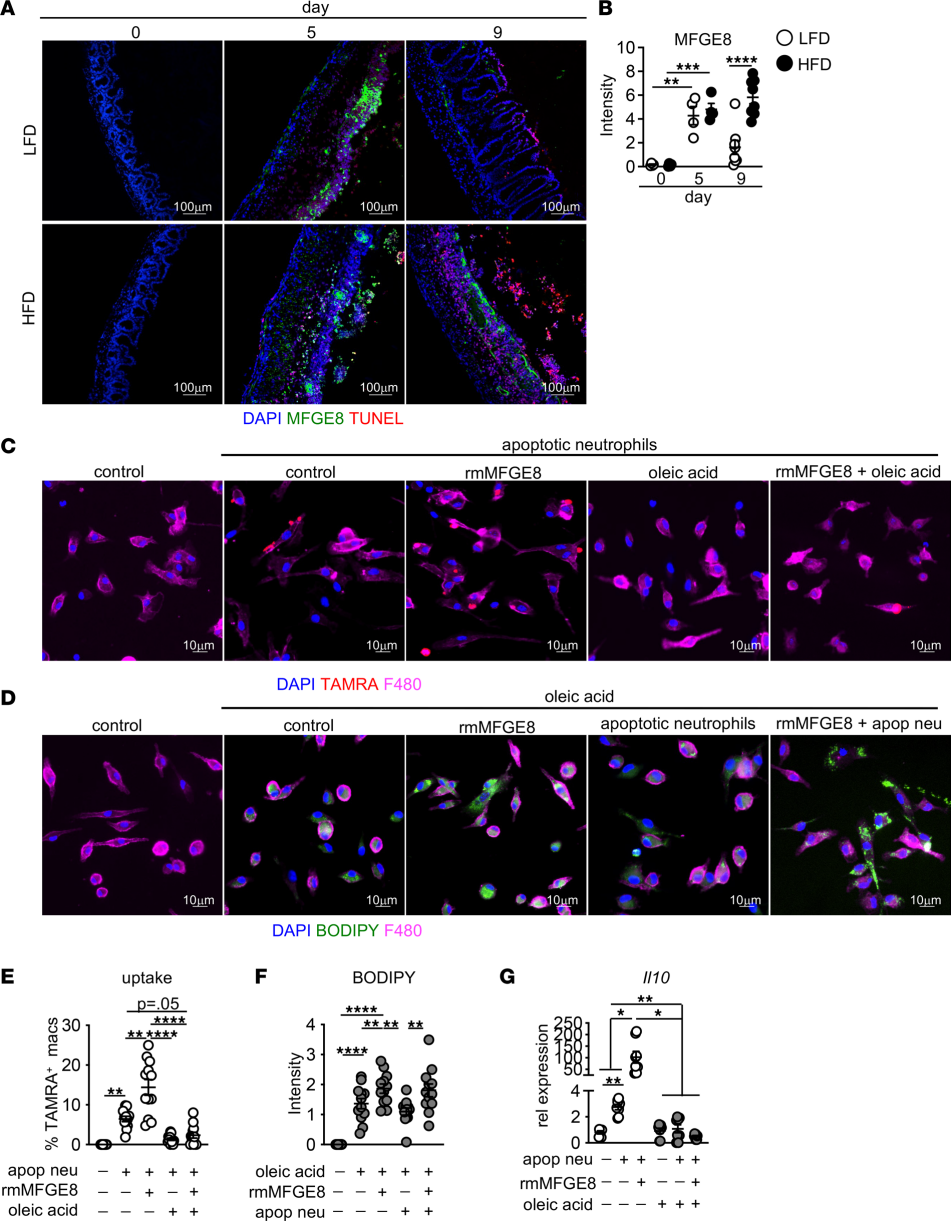
Dietary lipids impair MFGE8-mediated apoptotic neutrophil uptake. (**A** and **B**) Representative immunofluorescence staining and quantification for MFGE8 and TUNEL in the cecum of LFD- and HFD-fed mice at indicated day after DSS. Average of 3 HPF images (*n* = 4 D0 and D5, *n* = 8 D9) mice/group. (**C**) Representative images for TAMRA (apoptotic neutrophils) in control-treated BMDMs or BMDMs. (**D**) Representative images for BODIPY (lipid droplets) in control-treated BMDMs or BMDMs. (**E**) Quantification of TAMRA^+^ BMDMs in **C**. (**F**) Quantification of BODIPY in BMDMs in **D**. Data are representative of 2 experiments with 2 images per 3 technical replicates (**E** and **F**). (**G**) IL-10 gene expression in control- or oleic acid–treated BMDMs after exposure to apoptotic neutrophils alone or in the presence of rmMFGE8 (2 mg/mL). Two experiments were performed, with 4 technical replicates/group. Data are presented as mean ± SEM. *P < 0.05, **P < 0.01, ***P < 0.001, ****P < 0.0001. Statistical comparisons were performed using 1-way ANOVA with Tukey’s post hoc test (**B** and **E**–**G**), and if not indicated, a comparison is not significant. Scale bars: 100 μm or 10 μm, as indicated.

In the intestine, MFGE8 is constitutively expressed, with increased expression after intestinal damage that returns to baseline after tissue healing (56). Loss of MFGE8 results in delayed recovery after DSS treatment, but treatment with recombinant MFGE8 attenuates damage in colitis models (56, 57). We used IF staining to assess MFGE8 levels in LFD- and HFD-fed mice after DSS treatment (56). We found similar expression of MFGE8 in both groups before DSS treatment, with similar increases in response to DSS (Figure 10, A and B). However, by day 9, while expression decreased in the cecum of LFD-fed mice, MFGE8 remained elevated in HFD-fed mice (Figure 10, A and B). These findings demonstrate that cecal MFGE8 expression in HFD corresponded with impaired healing of damage.

To test if MFGE8 interactions with αVβ3 were required for uptake of both apoptotic cells and lipids, we pretreated BMDMs with RGD peptide, which blocks MFGE8 interaction with its receptor (25). This pretreatment reduced uptake of both apoptotic neutrophils and lipids (Supplemental Figure 4, F and G). This response was specific for apoptotic neutrophils and lipids, since RGD treatment of BMDMs did not reduce uptake of apoptotic thymocytes (Supplemental Figure 4H), which is Mertk dependent (58). These data demonstrate that macrophage uptake of both apoptotic neutrophils and dietary lipids requires MFGE8 and αVβ3.

Increased apoptotic neutrophils and MFGE8 expression in HFD-fed mice after injury suggested that MFGE8 may not facilitate apoptotic neutrophil clearance in the presence of dietary lipids. To test if there was competition between these 2 MFGE8 ligands, we first assessed whether macrophage uptake of apoptotic neutrophils or dietary lipids was enhanced by MFGE8. We exposed BMDMs to TAMRA-labeled apoptotic neutrophils or oleic acid in the presence or absence of recombinant mouse MFGE8 (rmMFGE8). The addition of rmMFGE8 increased uptake of both TAMRA-labeled apoptotic neutrophils and lipids, as assessed by BODIPY staining (Figure 10, C–F).

Since apoptotic neutrophils and oleic acid uptake both depend on MFGE8 and αVβ3, we asked if oleic acid inhibited uptake of apoptotic neutrophils. Coexposure of macrophages to apoptotic neutrophils and oleic acid resulted in decreased uptake of apoptotic neutrophils, with no impact on oleic acid uptake (Figure 10, C–F). Since we saw increased MFGE8 expression in the cecum of HFD-fed mice treated with DSS, we asked if rmMFGE8 could rescue neutrophil uptake in the presence of oleic acid. We treated BMDMs with apoptotic neutrophils, oleic acid, and rmMFGE8 and found that BMDM lipid uptake — but not apoptotic neutrophils — was increased (Figure 10, C–F). The findings suggest that MFGE8 favors uptake of lipids over apoptotic neutrophils, resulting in decreased macrophage uptake of dead cells when lipids are present.

We then assessed whether MFGE8 mediated macrophage IL-10 responses to apoptotic neutrophils. We exposed control-treated BMDMs, in the presence or absence of rmMFGE8, to apoptotic neutrophils and assessed IL-10 gene expression. As expected, IL-10 expression was increased in response to apoptotic neutrophils in control-treated BMDMs (Figure 10G). rmMFGE8 further increased *Il10* expression in BMDMs in response to apoptotic neutrophils (Figure 10G). In the presence of oleic acid, *Il10* expression was impaired in macrophages exposed to apoptotic neutrophils alone or in combination with rmMFGE8 (Figure 10G). These findings demonstrate that macrophage *Il10* in response to apoptotic neutrophils is dependent on MFGE8 and αVβ3 and is impaired in the presence of dietary lipids.

Taken together, our findings suggest that dietary lipids interfere with MFGE8 binding to apoptotic neutrophils, limiting their uptake with neutrophil accumulation pathological to the tissue. Furthermore, neutrophil uptake is required for induction of the antiinflammatory and prorepair cytokine IL-10. By interfering with MFGE8-mediated macrophage uptake of apoptotic neutrophils, exposure to dietary lipids sustains intestinal injury.

## Discussion

In this study, we demonstrate that acute exposure to HFD impairs recovery after intestinal damage, and we identified loss of a key resolving pathway after acute HFD treatment. We found increased weight loss and extended duration of tissue damage in the cecum, but not the distal colon, of mice acutely fed a HFD and exposed to DSS. This corresponds to the region of lipid accumulation and supports a direct effect of dietary lipids in preventing intestinal repair. Previous studies suggest that increased lipid consumption results in lipid deposition in the cecum and colon due to insufficient absorption in the small intestine (59, 60). Slow movement in the cecum could result in increased accumulation in cecum relative to the colon. The introduction of damage to this region could further prevent transport of lipids to the colon, resulting in increased lipid retention in the cecum, further amplifying defects in damage resolution.

In parallel, we also found accumulation of dead neutrophils within the cecum lamina propria and lumen of DSS-treated HFD-fed mice. Depletion of neutrophils in HFD-treated mice rescues from enhanced intestinal pathology. While neutrophils are important for clearance of microbes that penetrate the tissue after injury, excess neutrophils in tissues can also drive inflammation through their production of inflammatory mediators (61). Due to their short life-span, neutrophils then undergo cell death. If dead neutrophils are not cleared, they can undergo secondary necrosis, further amplifying tissue inflammation (7). Uptake of dead neutrophils activates tissue repair and antiinflammatory pathways in macrophages, including downregulation of chemokines that recruit neutrophils into tissue (6, 62, 63). We found that intestinal macrophages from HFD-fed mice had a decreased capacity to uptake dead neutrophils. We show that in vitro treatment of macrophages with oleic and palmitic acids, the primary lipids found in the HFD, limited macrophage uptake of dead neutrophils. Our results support direct interference by excess dietary lipids with a critical intestinal macrophage function necessary to promote the resolution of tissue damage. Continued intestinal neutrophil accumulation is a feature of IBD (5), and our studies suggest that defects in macrophage clearance of apoptotic neutrophils may serve as a contributing factor, especially in the context of HFD feeding.

Uptake and clearance of dead cells activate macrophage production of the antiinflammatory cytokine IL-10 (12, 13). Loss of IL-10 or IL-10 signaling corresponds with early-onset, severe human intestinal disease and with development of colitis in mouse models (64–66). IL-10 supplementation protects against DSS-induced colitis (67). While IL-10 is normally induced after DSS treatment, we do not find it upregulated in HFD-treated mice. Others have demonstrated that, in obesity, adipose tissue macrophages express decreased levels of IL-10 (18). Macrophage pretreatment with oleic and palmitic acids limits *Il10* expression after exposure to dead neutrophils. Overexpression of IL-10 rescued barrier repair defects and enhanced pathology in acute HFD-treated mice. Others have demonstrated that IL-10 directly supports epithelial proliferation in vitro (8). Importantly, overexpression of IL-10 does not protect against enhanced pathology after DSS treatment of HFD-fed mice, if epithelial cells lack IL-10Rα. We identified loss of IL-10 signaling downstream of dead neutrophil uptake after acute exposure to HFD.

In addition to its role in uptake, MFGE8 also suppresses neutrophil recruitment by downregulating neutrophil *Cxcr2* expression (68). We found elevated expression of *Cxcr2* in the cecum of HFD-fed mice after injury. Identifying whether dietary lipids also interfere with MFGE8-mediated downregulation of neutrophil *Cxcr2* expression would further provide insight into an additional mechanism by which dietary lipids promote increased inflammation.

MFGE8 levels are decreased in patients with ulcerative colitis (UC) compared with healthy controls and are associated with increased disease activity, highlighting the importance of MFGE8 in intestinal disease (69). However, increased MFGE8 levels in HFD-treated mice exposed to DSS does not improve tissue repair. Our study suggests that alterations in MFGE8 function may contribute to HFD-associated intestinal disease. We identified dietary lipid interference of MFGE8-mediated uptake of apoptotic neutrophils by macrophages as a mechanism by which dietary lipids impair resolution of intestinal injury. The 2 C-terminal discoidin domains of MFGE8 allow it to act as an opsin-like molecule that bind to the externalized PS on dead cells, while its N-terminal EGF domain binds to αvβ3 (24). Our work suggests that lipids outcompete PS for binding to the MFGE8 discoidin domains, preventing macrophage uptake of apoptotic neutrophils. This effect is specific for apoptotic neutrophils, as we found that lipids did not alter apoptotic thymocyte uptake, which others show depends on Mertk (58). MFGE8 likely has further roles in intestinal repair, since treatment with recombinant MFGE8 promotes epithelial migration to support wound closure in in vitro models (70). Lipid interference with MFGE8 binding to damaged epithelium may further contribute to lost repair. Better characterization of MFGE8 effector functions would allow for identification of ways to increase tissue repair and could be important therapeutically in resolving tissue damage in diet-associated diseases, such as atherosclerosis and intestinal disease.

We found that oleic acid, an unsaturated lipid, and palmitic acid, a saturated lipid, which both comprise the majority of the HFD, impair macrophage uptake of apoptotic neutrophils and *Il10* expression in response to apoptotic neutrophils. Dietary lipids consist of additional unsaturated lipids, including linoleic and palmitoleic acid and saturated lipids like stearic acid. Prior studies demonstrate that various lipid classes can impact macrophage anti- and proinflammatory functions, including cytokine production (71, 72). Understanding how various lipid classes, individual lipids, and the amounts of these lipids alter macrophage responses to injury, including uptake of apoptotic neutrophils, and intracellular pathways involved in downstream responses to injury, would provide better understanding of how the types of lipids we consume can prevent or predispose an individual to intestinal disease development. In future studies, it will be important to determine the impact of individual lipids on shared downstream pathways of macrophage breakdown of apoptotic neutrophils and dietary lipids on additional macrophage functions.

Recent data demonstrate that, in the steady state, intestinal macrophage and DC uptake of dead epithelial cells induces a homeostatic transcriptional program that promotes Tregs (73). It remains to be determined if the same PS receptors are utilized in this homeostatic pathway and, furthermore, if dietary lipids also interfere with dead cell clearance at steady state. Defects in similar clearance pathways are observed in several inflammatory disorders, and it will be important to understand whether dietary lipids interfere with dead neutrophil clearance in nonintestinal sites.

While diets high in fat have long been associated with human disease, these effects have been thought to be secondary to the expansion of adipose tissue, which supports systemic inflammation. Our results indicate that HFD not only increase inflammation after chronic exposure (74), but they also directly interfere with intestinal barrier repair. Unresolved damage leads to continued neutrophil recruitment into the tissue and increased microbial interaction with the epithelium, further potentiating tissue damage and limiting tissue repair. We have identified a mechanism of direct interference by dietary lipids in antiinflammatory and repair processes leading to sustained pathology, which may be of importance to human intestinal disease. Further study of this pathway may lead to new therapeutic targets to attenuate intestinal and other inflammatory diseases.

## Methods

### Experimental animals

Male C57BL/6J (stock no. 000664), CX_3_CR1-GFP/+ (stock no. 005582), CX_3_CR1-CreERT2 (stock no. 021160), and Lgr5-EGFP-IRES-creERT2 (stock no. 008875) mice were purchased from The Jackson Laboratory. IL-10 receptor α conditional (IL-10Rα^fl/fl^) mice were from Werner Muller (University of Manchester, Manchester, England) (75). Mice were kept and bred under standard specific pathogen–free (SPF) conditions at the Baylor College of Medicine or Memorial Sloan Kettering Cancer Center animal facility. All lines were backcrossed for at least 12 generations to the C57BL/6J background. All mouse experiments were performed with at least 4 mice per group in male mice between 6 and 8 weeks of age. Multiple experiments were combined to assess statistical significance. Littermate controls were used for each experiment, and mice were randomly assigned to experimental groups.

### Acute diet feeding and intestinal injury

Mice were fed 10% kcal LFD (Research Diets, D12450B) or 60% kcal HFD (Research Diets, D12492) ad libitum for 1 week prior to treatment with 2% DSS (Thermo Fisher Scientific, AAJ1448922) in drinking water for 5 days, followed by plain drinking water.

### Glucose tolerance test and insulin tolerance test

Glucose tolerance test (GTT) and insulin tolerance test (ITT) in mice fed LFD and HFD for 2 and 8 weeks were performed by the Mouse Metabolic Phenotyping Center at Baylor College of Medicine.

#### GTT.

After a 6-hour overnight fast, 1.5 g/Kg body weight of glucose was given i.p. to each mouse. Blood was collected from tail vein at 0, 15, 30, 60, and 120 minutes, and glucose levels were checked using a glucometer (Life Scan).

#### ITT.

In total, 1 U/kg body weight insulin (HUMULIN R) was injected i.p. to mice after a 4-hour fast. Blood glucose was measured as above.

### Cecal microbiota transplant

Mice were treated with a single dose of 20 mg/mL streptomycin (MilliporeSigma, S9137-25G) by per os (P.O.) with 1 g/1 L ampicillin (Fisher BioReagent, Thermo Fisher Scientific; BP176025) in drinking water for 2 weeks (76). Mice were switched to autoclaved water for 2 days before transplant of PBS-resuspended cecal content from mice fed HFD or LFD for 7 days.

### Histology

Ileum, cecum, proximal colon, and distal colon were fixed in Carnoy’s fixation for 1–2 days before being placed in methanol prior to paraffin embedding. Samples were deparaffinized, cut into 4 μM sections, and stained with hematoxylin or ABPAS. Images were taken with a Nikon Ti Eclipse or Leica microscope. Sections from 4–6 mice were used for blinded colitis scoring, according to established criteria (77, 78). The number of ABPAS^+^ goblet cells were counted per 10 villi for 5–9 mice per group. A score of 1 refers to mild mucosal inflammation. A score of 2 refers to inflammatory cell infiltrate in the mucosa and submucosa. A score of 3 consists of inflammatory infiltrates plus focal ulcerations. Focal ulceration with mucosa and submucosa inflammatory infiltrate comprises a score of 4. A score of 5 consists of extensive focal ulceration, in addition to mucosa and submucosa inflammatory infiltrate. A score of 6 is indicative of transmural inflammation and extensive ulcerations.

### IF tissue staining

Sections were fixed, embedded, deparaffinized, and cut as described above. Sections were permeabilized, blocked, and stained overnight at 4°C with the following primary antibodies at a 1:100 dilution: anti-Ki67 (polyclonal, Novus Biologicals, catalog NB110-89719), anti-OCLN (EPR20992, Abcam, catalog ab216327), anti-ZO1 (EPR19945-296, Abcam, catalog ab221547), anti-MUC2 (polyclonal, Cloud Clone, catalog PAA705Mu02), anti-Ly6g (1A8, BioXCell, catalog BE0075-1), anti-F4/80 (CI:A3-1, Abcam, catalog ab6640), and anti-MFGE8 (18A2-G10, MBL, catalog D199-3). Sections were washed, stained with the following secondary antibodies at a 1:200 dilution at room temperature for 1 hour: anti–rabbit NorthernLights-557 (R&D systems, catalog NL007), anti–rat Alexa Fluor 488 (Cell Signaling, catalog 4416), and anti–rabbit Alexa Fluor 488 (Cell Signaling, catalog 4412). This was followed by staining with DAPI (Sigma-Aldrich, catalog D9542) and mounting using Aqua Mount (Polysciences, catalog 18606-100) anti-fade mounting media before being cover slipped. In Situ Cell Death Detection Kit TMR red (TUNEL) (MilliporeSigma, catalog 12156792910) staining was performed according to manufacturer’s instructions prior to IF staining. FISH staining was performed prior to IF staining as described in ref. 79 using the following UNI519 universal primer-probe sequence: /5Alex594N/GTATTACCGCGGCTGCTG (Integrated DNA Technologies [IDT]). Images were taken on Nikon Ti Eclipse microscope using 20× and 100× objectives, and images were processed using FIJI.

### DNA preparation and 16S (v4) rRNA-Seq

Cecal DNA extraction was performed using a Promega Maxwell RSC PureFood GMO and Authentication Kit (AS1600) following manufacturer’s instructions. The library was generated following the protocol from the Earth Microbiome Project (http://press.igsb.anl.gov/earthmicrobiome/protocols-and-standards/16s/). Library quality and size verification was performed using PerkinElmer LabChip GXII instrument with DNA 1K Reagent Kit (CLS760673). Libraries were normalized to 2 nM and pooled using the same volume across all libraries. Pooled libraries were sequenced on the Illumina MiSeq with paired-end 250 using MiSeq Reagent Kit v2, 500-cycles (MS-102-2003). Demultiplexed raw reads were processed to generate an operational taxonomic unit (OTU) table using USEARCH version 11.0.667 (80). Taxonomic classification of OTU representative sequences was performed using usearch-sintax, an implementation of the SINTAX algorithm, version 16 of the Ribosomal Database Project (RDP) Training Set (81). The α diversity estimation was performed using the phyloseq R package (82). DNA preparation, sequencing, and analysis were performed by the JRI Microbiome Core at Weill Cornell Medicine.16S sequencing data are openly available in NCBI under BioProject ID PRJNA904807.

### Quantification of IF staining

#### Ki67 and ABPAS.

Three images were taken per mouse per group. For each image, Ki67^+^ or ABPAS^+^ cells per 10 crypts were counted and the average used for quantification.

#### MUC2 and FISH.

Three images from 4 mice per group were used to quantify MUC2 intensity and bacterial encroachment. For MUC2 intensity, ImageJ (NIH) was used to set a threshold and mask for each image, and pixel intensity was measured using ImageJ measuring tool. Bacterial encroachment was measured as the distance between the closets bacteria to the intestinal epithelium using the ImageJ measuring tool.

#### Total TUNEL^+^, total Ly6G^+^, and total Ly6G^+^TUNEL^+^ cells.

Four images were taken per mouse per group. Using ImageJ counting tool and the red (TUNEL) and blue fluorescent (DAPI) channels, the total number of cells positive for both DAPI and TUNEL were used to quantify the total number of TUNEL^+^ cells per image and the average count per mouse was used. Using an overlay of the red (TUNEL) and green channel (Ly6G), the number of double-positive cells were counted based on overlapping green and red fluorescence intensity using the ImageJ counting tool. Cells with only green fluorescence were considered live Ly6G^+^ neutrophils (Figure 4A).

#### MFGE8 intensity.

ImageJ was used to set a threshold and mask for each image, and pixel intensity was measured using ImageJ measuring tool.

#### Occludin and ZO1.

ImageJ was used to set a threshold and to mask an epithelial region for each image, and pixel intensity was measured using ImageJ measuring tool.

### BMDMs

BMDMs were differentiated from 8-week-old male and female C57BL/6J mice as previously described (83). Single-cell suspension of BM cells was cultured for 6 days in 50% DMEM (Corning, 10-017-CV) supplemented with 20% FBS (Corning, 35-10-CV), 30% L cell (ATCC CRL-2648) media, 2 mM glutamine (Hyclone, SH30034.01), 1 mM pyruvate (Thermo Fisher Scientific, 11360-070), 1 unit/mL pen/strep (Hyclone, SV30010), and 55 μM β-mercaptoethanol (Thermo Fisher Scientific, 21985023). Confirmation of macrophage differentiation was assessed by flow cytometry as described below. All assays were performed in DMEM supplemented with 10% FBS, 1 unit/mL pen/strep, and 1 mM HEPES (MilliporeSigma, H0887-100ml). iBMDMs from Jonathan C. Kagan (Harvard Medical School, Boston, Massachusetts, USA) were cultured as described (84).

### Dead cells

Neutrophils were isolated from BM using a density gradient (Histopaque 1077 and 1099) as previously described (85) and incubated for 24 hours at 37°C in DMEM containing 1% FBS. Thymocytes were treated with 1 mm staurosporine (Enzo, ALX-380-014-M001) for 4 hours at 37°C in DMEM containing 10% FBS. Death was assessed by trypan blue staining (Lonza, 17-942E). For phagocytosis assays, dead neutrophils and thymocytes were stained with TAMRA (Thermo Fisher Scientific, C1171) according to manufacturer’s instructions.

### Gene expression

RNA from whole cecum; 0.5 inches of the terminal ileum, proximal colon, and distal colon; or sorted macrophages were isolated using Trizol (Invitrogen, 15596018) according to manufacturer’s protocol. cDNA was synthesized using iScript reverse transcription kit (Bio-Rad, 1708841). Real-time quantitative PCR (qPCR) was performed using SYBR Green Supermix (Bio-Rad, 1725124) using a CFX384 Touch real-time PCR machine. Thermocycling program was 95°C for 2 minutes, followed by 40 cycles at 95°C for 15 seconds, 60°C for 30 seconds, and 72°C for 30 seconds. The following primers were used: mIL-10 forward (F): 5′-CCAGCTGGACAACATACTGCT-3′, mIL-10 reverse (R): 5′-AACCCCACAAGAGTTCTTTCAAA-3′; mGAPDH F: 5′-AATGTGTCCGTCGTGGATCT-3′, mGAPDH R: 5′-CATCGAAGGTGGAAGAGTGG-3′; mTNF F: 5′-TGGGAGTAGACAAGGTACAACCC-3′, mTNF R: 5′-CATCTTCTCAAAATTCGAGTGACA-3′; mOccludin F: 5′-TCAGGGAATATCCACCTATCACCTCAG-3′, mOccludin R: 5′-CATCAGCAGCAGCCATGTACTCTTCAC-3′; mZO1 F: 5′-AGGACACCAAAGCATGTGAG-3′, mZO1 R: 5′-GGCATTCCTGCTGGTTACA-3′; mCXCL1 F: 5′-TGAGCTGCGCTGTCAGTGCCT-3′, mCXCL1 R: 5′-AGAAGCCAGCGTTCACCAGA-3′; mCXCL2 F: 5′-GAGCTTGAGTGTGACGCCCCCAGG-3′, mCXCL2 R: 5′-GTTAGCCTTGCCTTTGTTCAGTATC-3′; and mCXCR2 F: 5′-TCT-GGC-ATG-CCC-TCT-ATT-CTG-3′, mCXCR2 R: 5′-AAG-GTA-ACC-TCC-TTC-ACG-TA-3′. Relative expression of target genes was determined using the ΔΔCT method with GAPDH used as an internal control.

### Phagocytosis and lipid uptake assay

Sorted intestinal macrophages isolated from LFD- and HFD-fed mice were incubated at a 1:2 ratio with TAMRA-loaded dead neutrophils for 1 hour in FACS tubes and cytospun before immunostaining. BMDMs were plated at 1 × 10^6^ cells in cover glass MakTek dish (MakTek Corporation, P35-1.5-14-C) or 1.5 × 10^6^ per well in 24-well tissue culture plates and incubated at a 1:2 ratio with TAMRA-labeled dead neutrophils, dead thymocytes, or FITC-labeled latex beads according to manufacturer’s instructions (Cayman Chemical, 500290) for 1 hour before RNA isolation or immunostaining. Using the ImageJ counting tool and automated cell counting described below, macrophages (F480, green) (Figure 7) that stained positive for TAMRA (apoptotic neutrophils) were used to quantify phagocytosis per image, and the average count and percentage was used for quantification. IL-10 gene expression was assessed as described above.

### Lipid treatment of BMDMs and BODIPY staining

Fatty acids were dissolved in ethanol (17). BMDMs were treated with 400 μm oleic acid (Nu-Chek Prep, U-46-A) or equivalent amount of solvent (ethanol) alone or with TAMRA-labeled dead neutrophils or thymocytes for 1 hour (17). To assess lipid uptake, BMDMs were exposed to 1 μm of BODIPY 493/503 (Invitrogen, D3922) for 30 minutes at room temperature after immunostaining. BODIPY intensity was measured using ImageJ.

### Recombinant MFGE8 and RGD peptide treatments

Apoptotic neutrophils or oleic acid were preincubated with 2 μg/mL of rmMFEG8 (R&D, 2805-MF-050) for 1 hour prior to addition to BMDMs. BMDMs were pretreated with 2 μg/mL of RGD peptide (Enzo Life Sciences, BML-P700) for 1 hour prior to exposure to apoptotic neutrophils or oleic acid.

### Automated cell counting

Microscopy pictures were processed with Fiji/ImageJ v.2.3.0/1.53f (86), and a custom macro was written to measure the fluorescence of single cells in each picture. Briefly, the macro detects single cells, extracts them from the main pictures, and measure the fluorescence signal for each individual channel. We first performed a background correction using the ballpoint background correction. Then, using representative pictures from an oleic acid–treated well, we set fluorescence thresholds for the detection of green (BODIPY), red (TAMRA), blue (DAPI), and magenta (F480) fluorescence (Figure 10). For cell detection, we used the magenta fluorescence channel to detect the cell silhouettes on every microscopy picture. Then we extracted individual silhouettes as rectangular frames from the main picture and removed the regions outside of the magenta area, to only leave cell-related fluorescence information. For each frame, we quantified the cell area (magenta signal), the number and size of cell nuclei (blue signal), red signals, and green signals. Finally, we summarized the overall fluorescence data in R v.4.1.2 (https://www.R-project.org/) and the tidyverse package (87). ImageJ macro and R script are available online at https://bitbucket.org/the-samuel-lab/mcalester-2022/

### Neutrophil depletion

Mice were injected i.p. with 400 μg of IgG2a isotype control (BioXCell, BE0089) or anti-Ly6g (BioXCell, BE0075-1) at day 4 of DSS treatment and continued every other day until the completion of the experiment.

### Lamina propria cell isolation

Isolation of lamina propria cells was performed as previously described (52, 88). In brief, indicated intestinal tissue was placed in PBS and was cut open, and luminal contents were removed. Intestine were cut in 1 cm sections and then treated with 1 mM DTT (MilliporeSigma, DN25) and 30 mM EDTA (Invitrogen, AM9261), followed by in 30 mM EDTA, both for 10 minutes at 37°C to remove mucus and epithelial cells. Tissue was then digested in 200 U/mL collagenase 8 (Sigma-Aldrich, C-2139) and 150 μg/mL DNase (MilliporeSigma, DN25) in RPMI supplemented with 10% FBS while shaking at 37°C for 1 hour, followed by separation on a 40%/80% Percoll (Cytiva, 17-0891-01) gradient.

### Flow cytometry and FACS

Flow cytometry and analysis were performed with an LSR II (BD Biosciences) and FlowJo software (Tree Star Inc.). Dead cells were excluded using the Live/Dead fixable aqua dead cell stain kit (Invitrogen). Macrophage populations were sorted on a FACSAria Cell sorter (BD Biosciences). Pooled samples from 5–7 CX_3_CR1-GFP/+ mice were used to obtain *n* = 1 for a total “*n*” equaling 4–5 for each group for gene expression and statistical analysis. Sorted macrophages from 1 mouse per group equaling *n* = 4–5 per group were treated with TAMRA-labeled dead neutrophils. The following antibodies were used for flow staining and or sorting: MHCII (M5/114.15.2, BioLegend, catalog 107620), CD11b (M1/70, BioLegend, catalog 101226), CD11c (N418, BioLegend, catalog 117317), Ly6C (AL-21, BD Pharmingen, catalog560525), CD45 (30-F11, BioLegend, catalog103149), and DAPI (Sigma-Aldrich, catalog D9542). We identified each population as follows: monocyte-derived macrophages (CX_3_CR1^hi^CD11b^+^MHCII^+^Ly6c^–^Tim4^–^), monocytes (CX_3_CR1^+^CD11b^+^Ly6C^+^), and conventional dendritic cells (CD11c^+^MHCII^+^CD103^+^).

### Overexpression of IL-10

Plasmid DNA expression of control and IL-10 (InVivoGen, puno1-mil10) were delivered i.v. at 10 μg DNA/mouse diluted in TransIT-EE Hydrodynamic Delivery solution (Mirus, MIR 5340) at 0.1 mL/g body weight 1 day after start of DSS treatment (89, 90).

### 4-hydroxy tamoxifen (4OHT) administration

4OHT (MillipporeSigma, 68392-35-8) was resuspended to 20 mg/mL in ETOH with heating to 37°C. 4OHT was diluted in corn oil (MilliporeSigma, 8001-30-7), and mice were injected i.p. with 0.2 mg every 3 days (CX_3_CR1-CreERT2 and control) starting on day 0 of DSS treatment or on 2 sequential days 1 week before the start of DSS treatment (LGR5-CreERT2 and control).

### Statistics

One-way ANOVA with Tukey’s post hoc test or unpaired 2-tailed Student’s *t* test was performed using a 95% CI. All data are presented as mean ± SEM. All analyses were performed using GraphPad Prism version 8.0. Differences were considered to be significant at *P* values of less than 0.05.

### Study approval

All experiments were performed in accordance with approved protocols by the IACUC at Baylor College of Medicine and Memorial Sloan Kettering Cancer Center.

## Author contributions

GED and AAH designed experiments and wrote the manuscript with input from all coauthors. AAH, MK, DFZR, and GED performed, designed, and analyzed the experiments. AMM, LCC, HWS, MCR, WJHW, KN, and JMH performed experiments. RSL supervised 16s sequencing and designed experiments. AAH, AA, and BSS designed the automated counting script. AAH is the primary lead on the project and is listed first as a co–first author.

## Supplementary Material

Supplemental data

## Version 1 12/20/2022

In-Press Preview

## Version 2 02/08/2023

Electronic publication
