# Supplementary material for: Acute high-fat diet impairs macrophage-supported intestinal damage resolution
Source: JCI Insight. 2023 Feb 8;8(3):e164489. doi: 10.1172/jci.insight.164489 (PMC9977439; doi:10.1172/jci.insight.164489)
Supplement: Supplemental data [file jciinsight-8-164489-s187.pdf]

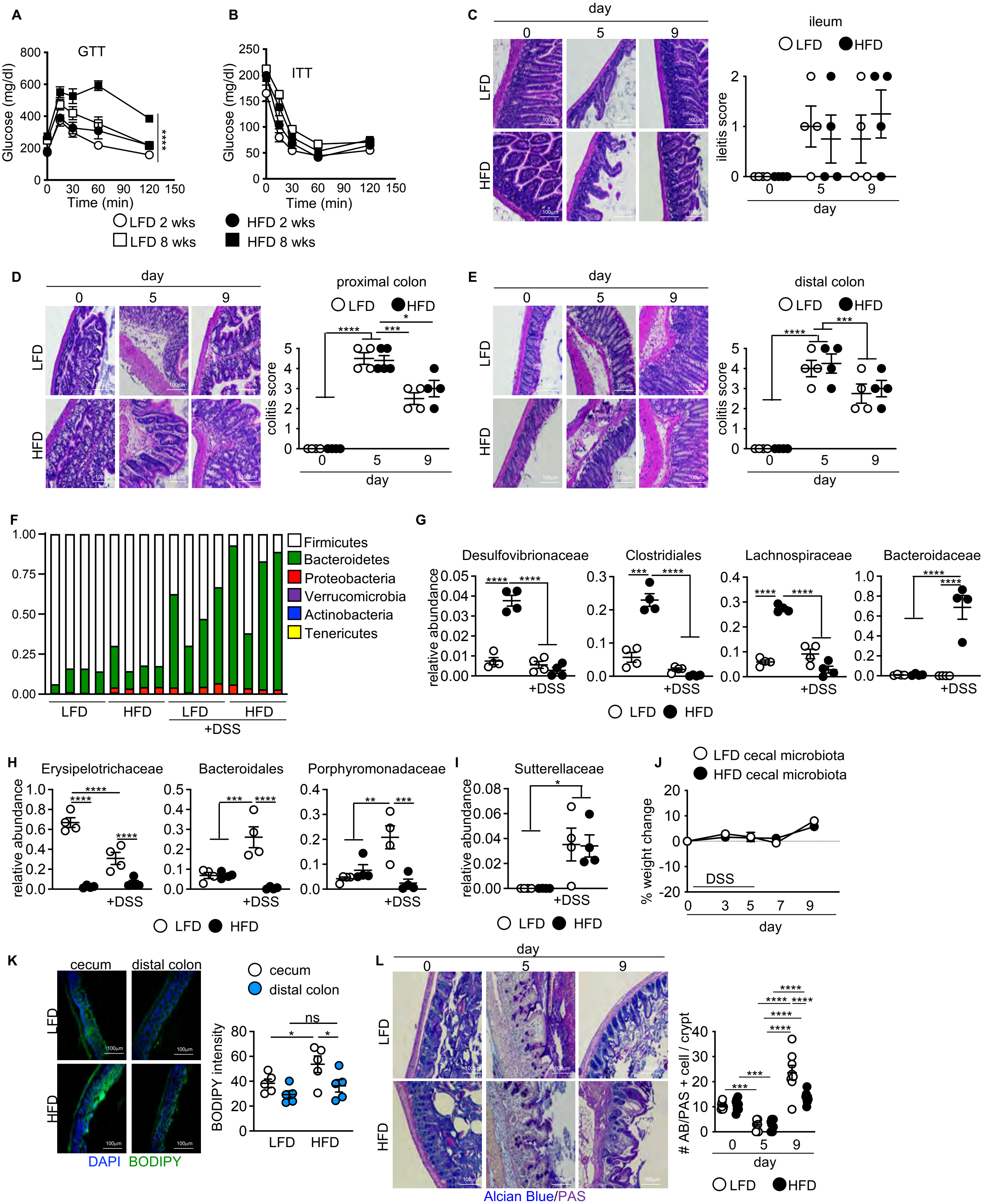

Supplemental Figure 1. Short-term HFD feeding does not lead to major metabolic changes or increased damage in ileum or colon after DSS treatment but alters microbial composition and lipid content in the cecum. (**A-B**) Glucose tolerance test and insulin tolerance test of mice treated with LFD or HFD for 2 or 8 weeks (n= 4 to 5 mice/group). (**C-E**) Representative H&E staining and pathology scores of the ileum, proximal colon, and distal colon of LFD and HFD fed mice. (**F-I**) 16s sequencing of cecal contents from LFD and HFD mice before and after DSS treatment (n= 4 mice/group). **(J)** Weightloss after DSS treatment in WT mice transplanted with cecal contents from LFD or HFD donor mice (n = 5 mice/group) **(K)** Quantification and representative images of BODIPY staining in cecum and distal colon of LFD and HFD mice before DSS treatment (n = 5 mice/group). (**L**) Representative image and quantification of Alcian Blue/PAS staining for goblet cells at indicated day post DSS (dark blue, (n = 8 mice/group)). Scale bar equals 100 microns. Data shown are representative of two experiments. Data are presented as mean ± SEM,. *P<.05, **P<.01, ***P<.001,****P<.0001 (Student t test, One-way Anova or Two-way Anova) and if not indicated, a comparison is not significant.

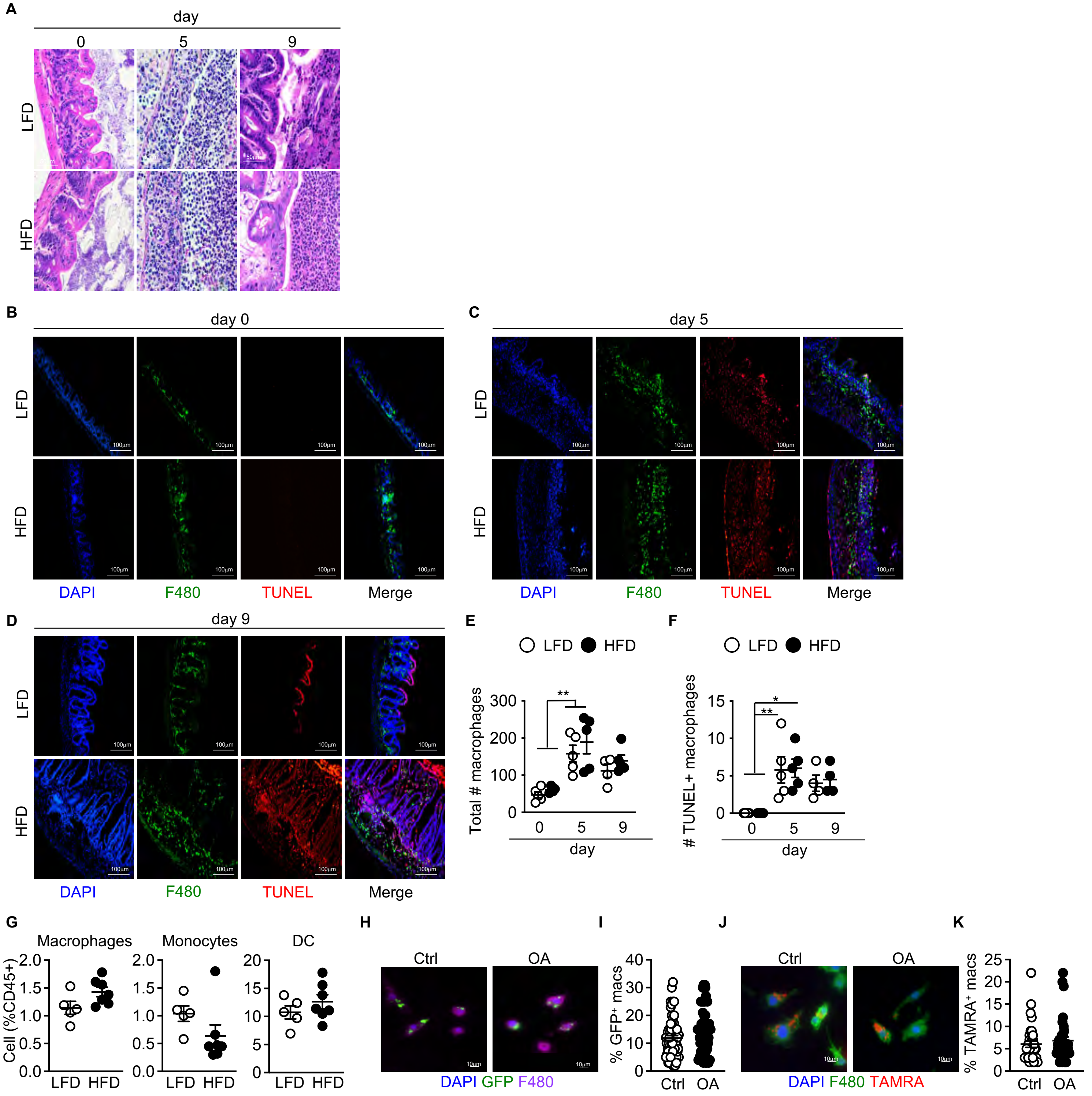

Supplemental Figure 2. Tissue macrophages increase after DSS challenge similarly in the cecum of LFD and HFD mice and lipids do not alter macrophage uptake of beads or apoptotic thymocytes. (**A**) Representative H&E staining showing neutrophil infiltration in LFD and HFD fed control and DSS treated mice (n = 4-5 mice/group). (**B-D**) Representative immunofluorescence staining for F4/80 (green), nuclei (Dapi, blue), and dead cells (TUNEL, red) in LFD and HFD DSS treated mice on day 0, 5 and 9 of DSS treatment (n = 4/group). Scale bar equals 100 microns. (**E**) Total (n = 4/group) and (**F**) dead macrophage numbers in LFD and HFD treated mice at day 0, 5, and 9 of DSS treatment. (n = 4/group). (**G**) Flow cytometry analysis of CX_3CR1+ macrophages and monocytes and CD103+ dendritic cells in the cecum of LFD and HFD DSS mice (n = 7 mice/group). (**H-I**) Immunofluorescence staining and quantification of percentage of TAMRA+ control (Ctrl) or oleic acid (OA) treated macrophages after exposure to (**H**) GFP labeled latex beads (n = 6 images/group, 3 experiments) or (**I**) dead thymocytes (n = 6 images/group, 3 experiments). Data shown are representative of three experiments. Data are presented as mean ± SEM. Scale bar equals 100 and 10 microns. *P<.05, **P<.01 . Statistical significance was analyzed by Student *t* test and if not indicated, a comparison is not significant.

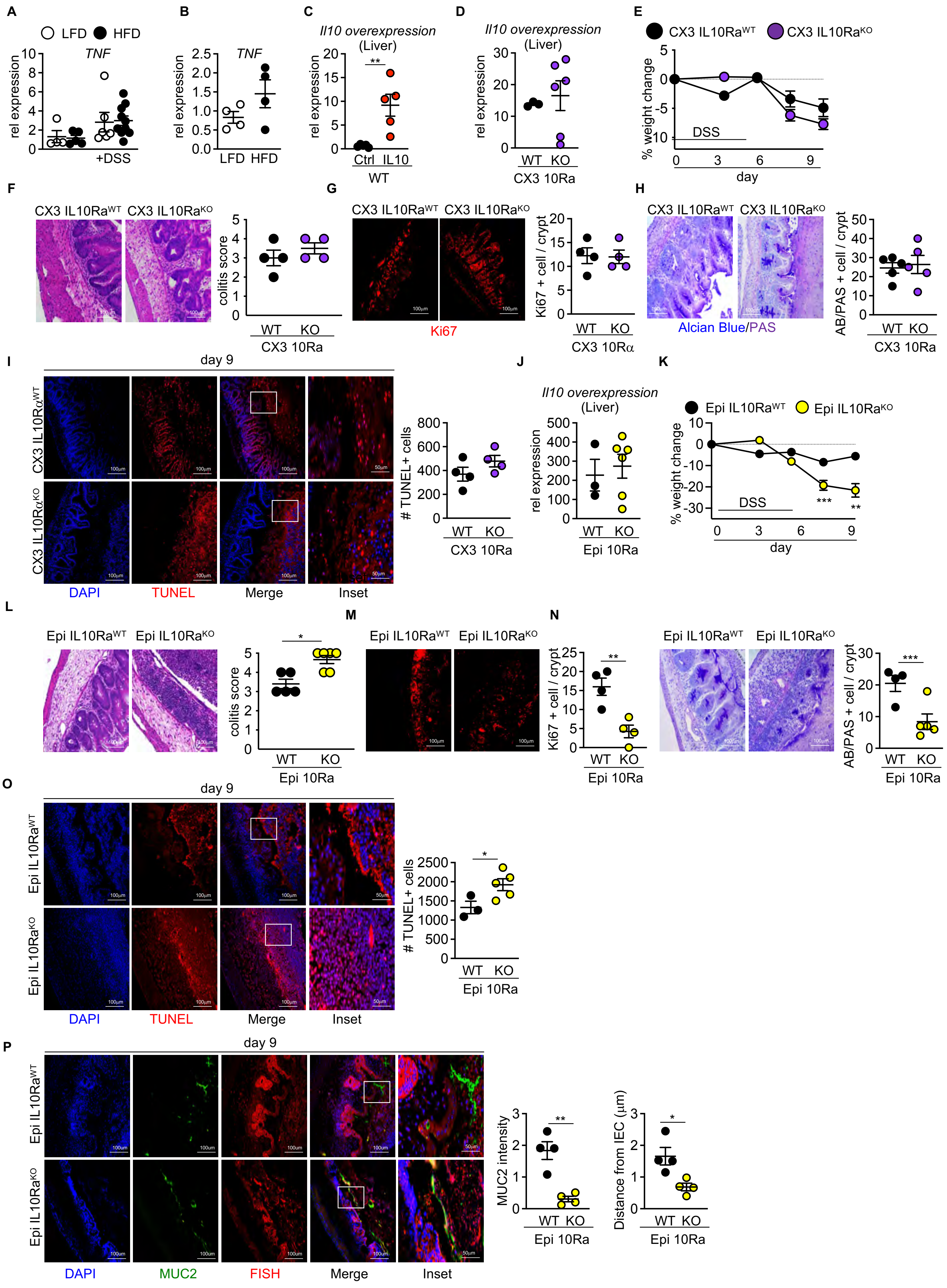

A
LFD HFD
TNF
rel expression
+DSS
B
TNF
rel expression
LFD HFD
C
Il10 overexpression (Liver)
rel expression
**
Ctrl IL10
WT
D
Il10 overexpression (Liver)
rel expression
WT KO
CX3 10Ra
E
CX3 IL10Ra^WT CX3 IL10Ra^KO
% weight change
DSS
day
F
CX3 IL10Ra^WT CX3 IL10Ra^KO
colitis score
WT KO
CX3 10Ra
G
CX3 IL10Ra^WT CX3 IL10Ra^KO
Ki67
Ki67 + cell / crypt
WT KO
CX3 10Rα
H
CX3 IL10Ra^WT CX3 IL10Ra^KO
Alcian Blue/PAS
AB/PAS + cell / crypt
WT KO
CX3 10Ra
I
day 9
CX3 IL10Rα^WT
CX3 IL10Rα^KO
DAPI TUNEL Merge Inset
TUNEL+ cells
WT KO
CX3 10Ra
J
Il10 overexpression (Liver)
rel expression
WT KO
Epi 10Ra
K
Epi IL10Ra^WT Epi IL10Ra^KO
% weight change
DSS
***
**
day
L
Epi IL10Ra^WT Epi IL10Ra^KO
colitis score
*
WT KO
Epi 10Ra
M
Epi IL10Ra^WT Epi IL10Ra^KO
N
Ki67 + cell / crypt
**
WT KO
Epi 10Ra
Epi IL10Ra^WT Epi IL10Ra^KO
AB/PAS + cell / crypt
***
WT KO
Epi 10Ra
O
day 9
Epi IL10Ra^WT
Epi IL10Ra^KO
DAPI TUNEL Merge Inset
TUNEL+ cells
*
WT KO
Epi 10Ra
P
day 9
Epi IL10Ra^WT
Epi IL10Ra^KO
DAPI MUC2 FISH Merge Inset
MUC2 intensity
**
WT KO
Epi 10Ra
Distance from IEC (μm)
*
WT KO
Epi 10Ra

Supplemental Figure 3. Short-term HFD feeding does not increase inflammatory cytokines and IL10Ra on epithelial cells, but not macrophages, is required for IL10 mediated protection from intestinal damage after HFD feeding. (**A**) Cecum gene expression of TNF in LFD and HFD fed control and DSS treated mice (n= 4-5 per group). (**B**) Gene expression of *TNF* in sorted intestinal macrophages from LFD and HFD fed DSS treated mice (n= 4 per group) for sorted cells gene expression where sorted cells from 5 mice pooled per "n" per group. (**C**) Liver IL10 gene expression in WT mice treated with hydrodynamic delivery of a control (Ctrl) or IL10–producing plasmid (n= 4 per group). (**D**) Liver IL10 gene expression in tamoxifen treated littermate CX3 IL10Ra^{WT} and CX3 IL10Ra^{KO} HFD DSS mice with hydrodynamic delivery of an IL10–producing plasmid. (**E**) Weight loss in tamoxifen treated littermate CX3 IL10Ra^{WT} and CX3 IL-10Ra^{KO} mice treated with HFD and DSS and hydrodynamic delivery of an IL10–producing plasmid (n= 4 per group). The following panels are measurements in cecum of mice in panel (E). (**F**) Representative images and colitis score. Representative imaging and quantification of (**G**) proliferating cells and (**H**) goblet cells. (**I**) Representative imaging and quantification of TUNEL positive cells. (**J**) Liver IL10 gene expression in tamoxifen treated Epi IL10Ra^{WT} and Epi IL10Ra^{KO} HFD DSS mice treated with hydrodynamic delivery of an IL10–producing plasmid (n= 4 per group). (**K**) Weight loss in tamoxifen treated Epi IL10Ra^{wt} and Epi IL10Ra^{KO} mice treated with HFD and DSS and hydrodynamic delivery of an IL10–producing plasmid (n = 5-6 mice/group). The follow panels are measurements in cecum of mice in panel (K). (**L**) Representative H&E image and blinded colitis score. Representative staining and quantification for (**M**) proliferating cells (Ki67^{+}) and (**N**) goblet cells. (**O**) Representative immunofluorescence and quantification of TUNEL^{+} cells. (**P**) Representative image stained for nuclei, mucus, and bacteria and quantification of muc2 and bacterial encroachment. Scale bar equals 100 microns. Data shown are representative of two experiments. Data are presented as mean ± SEM,. *P<.05, **P<.01, ***P<.001 (Student t test) and if not indicated, a comparison is not significant.

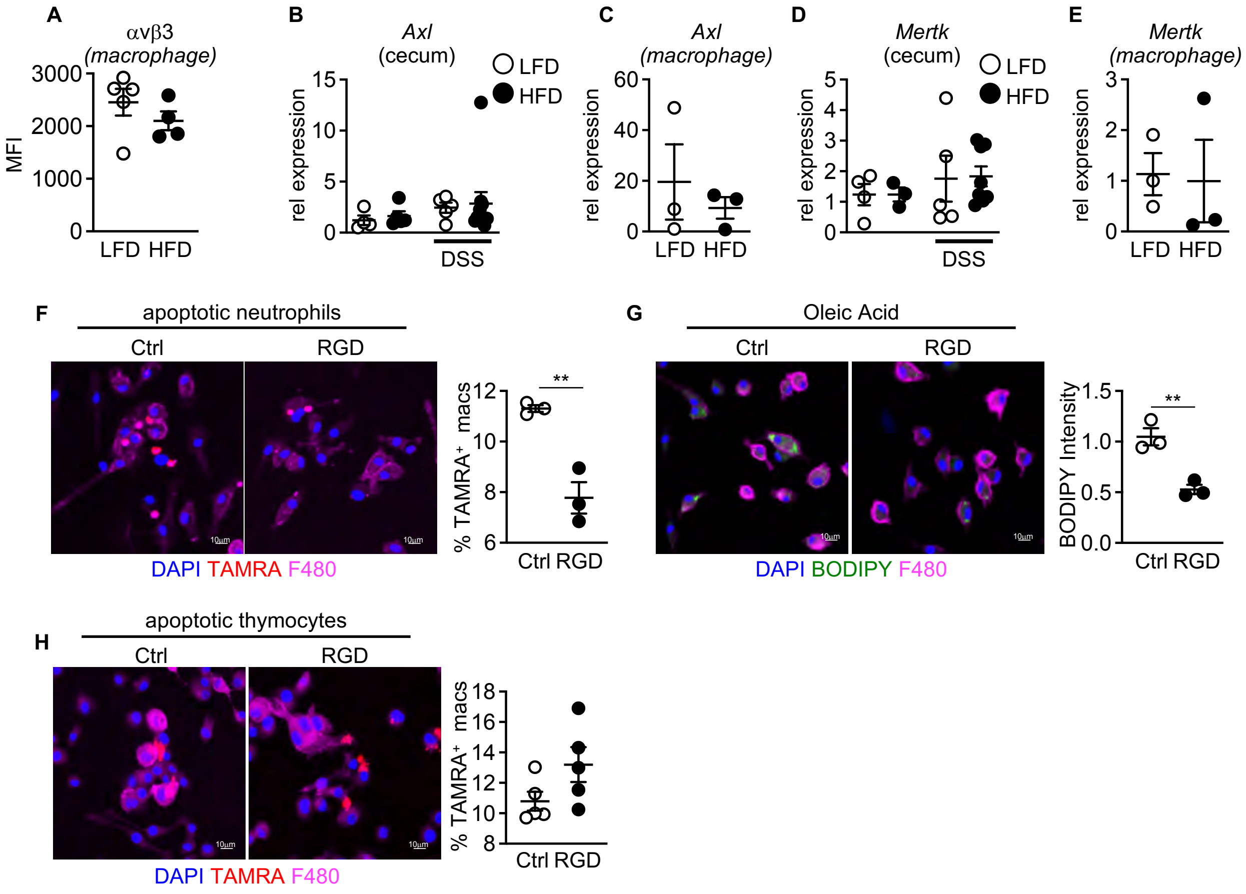

Supplemental Figure 4. HFD feeding does not change macrophage phagocytosis receptor expression and RGD peptide blocks uptake of apoptotic neutrophil and oleic acid, but not apoptotic thymocytes. (**A**) Mean fluorescent intensity of surface expression of $\alpha v\beta 3$ on intestinal macrophages in LFD and HFD fed DSS treated mice. (**B**) *Axl* gene expression in cecum of LFD and HFD fed mice before and after DSS treatment. (**C**) *Axl* gene expression sorted intestinal CX_3CR1^+ macrophages from LFD and HFD fed mice after DSS treatment. (**D**) *Mertk* gene expression in cecum of LFD and HFD fed mice before and after DSS treatment. (**E**) *Mertk* gene expression in sorted intestinal CX_3CR1^+ macrophages from LFD and HFD fed DSS treated mice (n= 4-10 mice/group.) (**F-G**) Representative imaging and quantification of BMDMs pre-treated with 2 ug/ml of RDG peptide for 1 h and then exposed to (**F**) TAMRA-labeled apoptotic neutrophils or (**G**) oleic acid. (**H**) Representative imaging and quantification of BMDMs pre-treated with 2 mg/ml of RDG peptide for 1 h and then exposed to TAMRA-labeled thymocytes. Scale bar 10 microns. Data shown are representative of two experiments. Data are presented as mean ± SEM,. **P<.01 (Student t test) and if not indicated, a comparison is not significant.
